# Bacteriophage–prokaryote dynamics and interaction within anaerobic digestion processes across time and space

**DOI:** 10.1186/s40168-017-0272-8

**Published:** 2017-05-31

**Authors:** Junyu Zhang, Qun Gao, Qiuting Zhang, Tengxu Wang, Haowei Yue, Linwei Wu, Jason Shi, Ziyan Qin, Jizhong Zhou, Jiane Zuo, Yunfeng Yang

**Affiliations:** 1 State Key Joint Laboratory of Environmental Simulation and Pollution Control, School of Environment, Tsinghua University, Beijing, 10084 China; 2 Institute for Environmental Genomics, Department of Microbiology and Plant Biology and School of Civil Engineering and Environmental Sciences, University of Oklahoma, Norman, OK 73019 USA; 3 Earth and Environmental Sciences Division, Lawrence Berkeley National Laboratory, Berkeley, CA 94270 USA

**Keywords:** Microbiome, Bacteriophages, Anaerobic digestion, Time dynamics, GeoChip

## Abstract

**Background:**

Bacteriophage–prokaryote dynamics and interaction are believed to be important in governing microbiome composition and ecosystem functions, yet our limited knowledge of the spatial and temporal variation in phage and prokaryotic community compositions precludes accurate assessment of their roles and impacts. Anaerobic digesters are ideal model systems to examine phage–host interaction, owing to easy access, stable operation, nutrient-rich environment, and consequently enormous numbers of phages and prokaryotic cells.

**Results:**

Equipped with high-throughput, cutting-edge environmental genomics techniques, we examined phage and prokaryotic community composition of four anaerobic digesters in full-scale wastewater treatment plants across China. Despite the relatively stable process performance in biogas production, phage and prokaryotic groups fluctuated monthly over a year of study period, showing significant correlations between those two groups at the α- and β-diversity levels. Strikingly, phages explained 40.6% of total variations of the prokaryotic community composition, much higher than the explanatory power by abiotic factors (14.5%). Consequently, phages were significantly (*P* < 0.010) linked to parameters related to process performance including biogas production and volatile solid concentrations. Association network analyses showed phage–prokaryote pairs were shallowly conserved since they were detected only within small viral clades.

**Conclusions:**

Those results collectively demonstrate phages as a major biotic factor in controlling prokaryotic composition and process performance. Therefore, phages may play a larger role in shaping prokaryotic community dynamics and process performance of anaerobic digesters than currently appreciated.

**Electronic supplementary material:**

The online version of this article (doi:10.1186/s40168-017-0272-8) contains supplementary material, which is available to authorized users.

## Background

The composition of microbiome is modulated by both biotic and abiotic factors, but knowledge on biotic control is extremely scarce, owing to the lack of appropriate tools to explore biological interactions. Recently, bacteriophages are incrementally recognized as the most abundant biological entities on earth, estimated to be ten phages for every bacterial cell in most ecosystems [1–3]. Therefore, there has been mounting interest to assess the importance of phage predation on microbial population dynamics [2, 4]. In the oceanic environment, phages are responsible for lysing over 25% of microbial cells [2]. In natural freshwater environment, phage lysis is responsible for up to 71% of microbial mortality, which fundamentally affects microbial food webs and aquatic carbon cycling [3]. Virus-mediated recycling of organic matter, termed “viral shunt,” has been shown to regulate global carbon and nitrogen cycles [5, 6].

We speculate that bacteriophages are important in shaping microbiomes in anaerobic digesters of wastewater treatment plants (WWTPs) and have been domesticated to produce desired effects, that is, to reduce the volume and mineralize organic matter into biogas before discharging activated sludge of WWTPs into the environment. Although biotic and abiotic factors controlling microbiome are essential to understanding such processes [7], little is known about top-down control through phage lysis, an issue considered, thus far, in only a handful of studies [1, 8–10]. In WWTPs, viral concentrations have been reported to be 10^8^–10^9^ virus-like particles/milliliter, which is higher than any other ecosystem studied to date [1]. In addition, the relatively stable, nutrient-rich conditions in WWTPs make the systems ideal hunting grounds for phages to attack microbes.

Our hypothesis that phage–host dynamics and interaction affect prokaryotic community composition and process performance of anaerobic digesters is the basis for this study. In four anaerobic digesters of full-scale WWTPs located in geographic distant cities of China (the capital city of Beijing, Qingdao City of Shandong Province, and Ningbo City of Zhejiang Province), we followed changes in prokaryotic community composition by 16S rRNA gene amplicon sequencing over consecutively 12 months. A variety of selected phages were detected by GeoChip, a microarray-based tool with high accuracy in quantification [11, 12]. Aiming to understand phage–host dynamics in this complex environment, we hypothesize that prokaryotic community diversity and/or composition in anaerobic digesters are significantly controlled by phages. Given the central role of bacteria and archaea for the stepwise anaerobic digestion process to hydrolyze organic substrates into smaller molecules such as volatile fatty acids (VFA), hydrogen, and methane, we further hypothesize that phage dynamics are linked to process performance. By in-depth examination of the relationship between individual phages and prokaryotic OTUs, we aim to unveil the life strategy of phages and their interaction with prokaryotic hosts.

## Results

### Dynamics of phages and prokaryotes as functions of time and space

A total of 56,143 prokaryotic OTUs and 183 phage genes belonging to 78 phages were detected from 48 samples. The α-diversity of phages showed considerable fluctuation over time, with generally higher α-diversity at Ningbo than others (Additional file 1: Figure S1). The overall composition of phages and prokaryotes were distinct (*P* < 0.05) among samples from Beijing, Qingdao, and Ningbo anaerobic digesters maintained at mesophilic temperature (Ningbo-M) and thermophilic temperature (Ningbo-T), as examined by complementary non-parametric dissimilarity tests (Mrpp, Anosim, and Adonis) (Additional file 1: Table S1).

About half of phage communities (91 out of 183 phages) were comprised of “common” phages across the four digesters (Beijing, Qingdao, Ningbo-M, and Ningbo-T), while 45 phages were only detected in one digester (Additional file 1: Figure S2). Phages present in all digesters were averagely 2.4 folds in relative abundance of those present only in one digester, suggesting that abundant phages tended to be universally present in different locations. Within an anaerobic digester, there were large temporal variations for individual phages (Additional file 1: Figure S3). For example, one of the most abundant phages in Beijing samples, *Enterobacteria* phage RB14, oscillated averagely by 48.9% in relative abundance. By contrast, another abundant phage in Beijing samples, *Enterobacteria* phage RB43, oscillated only by 17.3% over time.

### Top-down control of phages on prokaryotic communities

**Fig. 1:**
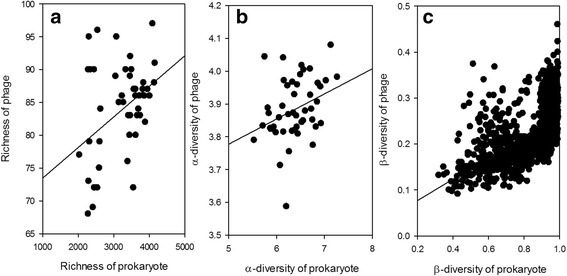
Spearman correlations between richness (**a**), α-diversity (**b**), and β-diversity of phage and prokaryotes (**c**). The significance of correlation was determined by *P* < 0.050

To better understand phage–prokaryote dynamics, we examined whether there was any relationship between prokaryotes and phages. The richness of prokaryotic OTU and phage were weakly but significantly correlated (*R* = 0.301, *P* = 0.037) (Fig. 1a). Similar result was observed in α-diversity of prokaryotic OTUs and phages (*R* = 0.322, *P* = 0.026) (Fig. 1b). By contrast, much stronger correlation was observed between prokaryotic OTU and phage β-diversity (Bray-Curtis) (*R* = 0.674, *P* < 0.001) (Fig. 1c).

We carried out multiple regression matrix (MRM) to explore the influence of biotic and abiotic factors on prokaryotic community composition. Our results showed that phages, as biotic factors, explained 40.6% of total variations of prokaryotic community composition (Table 1), much higher than the explainable power of abiotic factors altogether (14.5% for COD, TS, VS, pH, Fe, Cu, Cr, Cl^−^ and SO_4_^2−^), demonstrating that phages were essential in shaping prokaryotic communities.

**Table 1:** Influence of abiotic and biotic factors on prokaryotic community composition by multiple regression matrix (MRM)

Influence factors	*R* ^*2*^	*P*
Abiotic factors^a^	0.1452	0.001
Biotic factors^b^	0.4060	0.001

^a^Abiotic factors: COD, TS, VS, pH, Fe, Cu, Cr, Cl^−^, and SO_4_^2−^

^b^Biotic factors: phage community composition

### The impact of phages on process performance

**Fig. 2:**
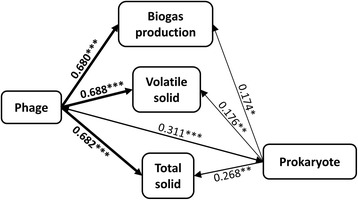
Effects of phages or prokaryotes on performance processes. The *number* on the arrow is the coefficient of determination (*R* ^*2*^). The significance is determined by partial least squares model. ***P < 0.001, **P < 0.010, *P < 0.050

Measurements of process parameters were summarized in Additional file 1: Table S2. To better integrate these complicated interrelationships, we constructed a partial least squares path model (PLS-PM) relating phages and prokaryotic OTUs to process parameters (Fig. 2), which allowed us to explore the cause and effect relationships among observed (indicators) and latent (constructs) parameters. Phages showed strong effects on biogas production (*R* ^*2*^ = 0.680, *P* < 0.001) and volatile solid concentrations (*R* ^*2*^ = 0.688, *P* < 0.001), demonstrating that the phage community was an important but previously overlooked factor affecting process performance of anaerobic digesters. Prokaryotic OTUs also showed significant effects on process parameters, albeit to a lesser extent on biogas production and volatile solid concentrations.

**Fig. 3:**
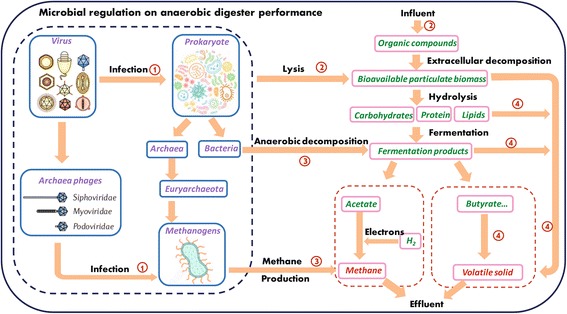
The biochemical pathway analysis to link phage and microbial communities to process performance of anaerobic digesters. Microbial communities are represented in *purple*. Intermediate metabolites are represented in *green*. Methane and volatile solid, which are process performance indices, are represented in *red*. Pathways are shown as *arrows*

**Fig. 4:**
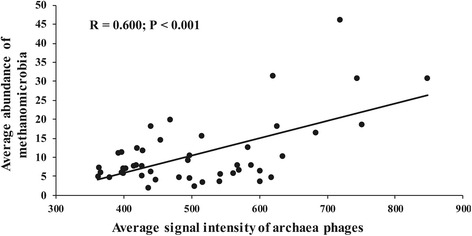
Correlation between average signal intensity of archaea phage genes and average abundance of phylum *Euryarchaeota* (*N* = 46)

A biochemical pathway analysis was further carried out to link phage and microbial communities to process performance of anaerobic digesters. Four important pathways were included (Fig. 3). Pathway ① showed the infection of phages on prokaryotic hosts. It has been shown that phage families *Myoviridae*, *Siphoviridae*, and *Podoviridae* specifically infect a major archaea phylum *Euryarchaeota* [13, 14]. Consistently, average gene signal intensity of phage families *Myoviridae*, *Siphoviridae*, and *Podoviridae* significantly correlated (*R* = 0.600, *P* < 0.001) with the average abundance of Phylum *Euryarchaeota* (Fig. 4). A closer examination showed that all of the *Euryarchaeota* OTUs detected in our samples belonged to methanogenic class *Methanomicrobia* (Additional file 1: Table S3), revealing a linkage between phages and methane production. Pathway ② showed that the lysis of prokaryotes by phages, known as viral shunt, increased organic matter supply in anaerobic digesters, which resulted in a positive feedback of net primary productivity [15–17]. Pathway ③ showed that organic matters from the influent of digesters or lysis of microbes were converted into acetate by anaerobic fermentation and subsequently methane by methanogenesis. Pathway ④ showed that the organic substrates that were not decomposed by microbes became volatile solids in effluent of anaerobic digesters.

### The network analysis between phages and prokaryotes

**Fig. 5:**
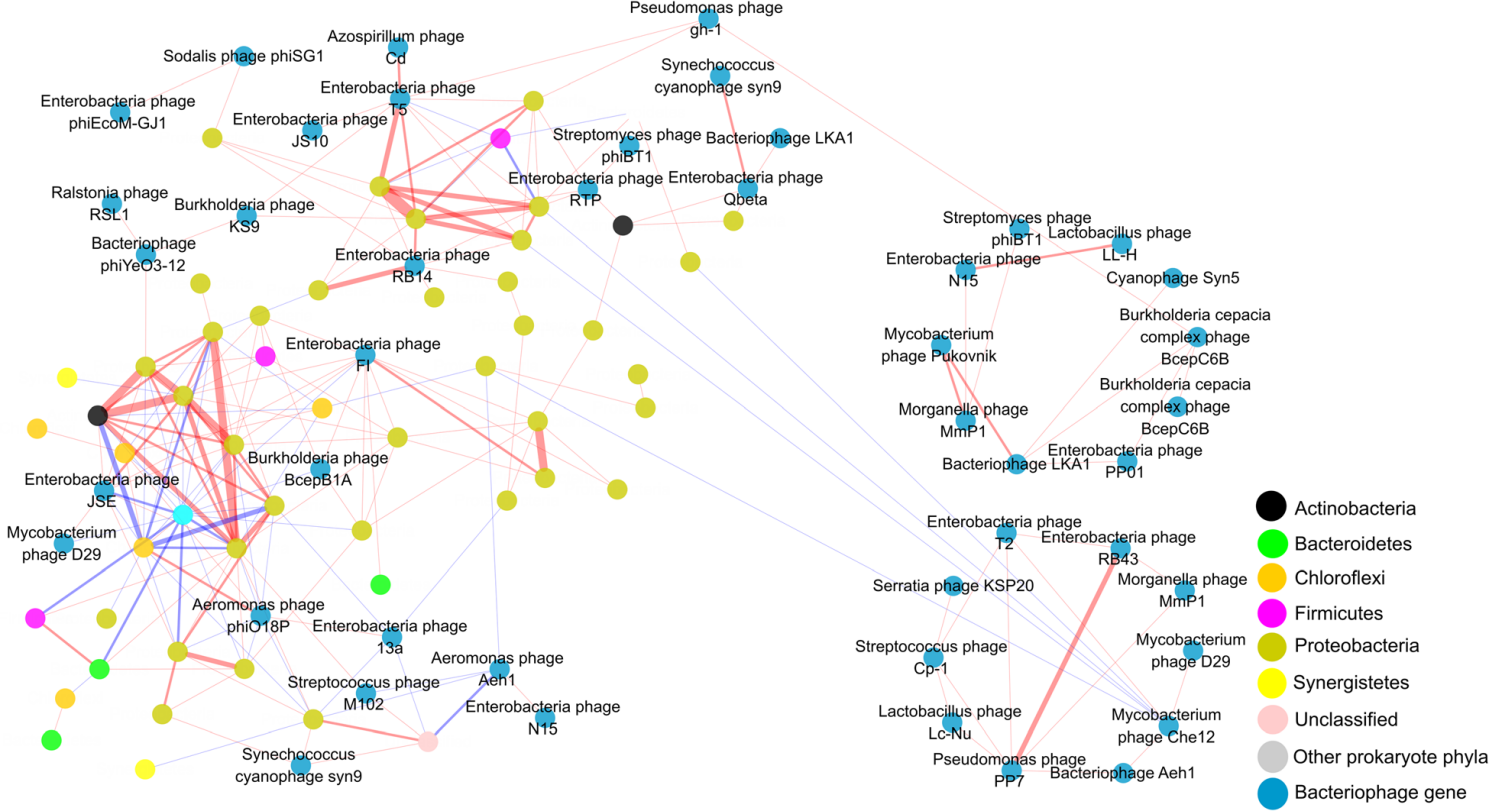
The association network comprised of phage and prokaryotic OTUs. Modules with equal or less than five nodes are omitted. The positive or negative linkages of the association networks are based on positive or negative Spearman’s correlations between any pairs of nodes. Positive linkages are shown in *red*, while negative linkages are shown in *blue*. Spearman’s correlation coefficients are indicated by *line width*

An association network based on Spearman’s correlations was constructed to deconvolute complex relationships between phage and prokaryotes (Fig. 5). Although prokaryotic OTUs greatly outnumbered detected phages, the network generated from random matrix theory-based algorithm only contained 55 prokaryotic OTUs and 49 phages, suggesting that their interactions were strong and persistent. The network had general topological features of scale-free (power-law *R* ^*2*^ of 0.753), small-world (average path distance of 4.888), and hierarchical (average clustering coefficient of 0.294) (Additional file 1: Table S4). It also showed a modular structure (modularity of 0.570), which were typical of biological networks [18, 19].

Half of the links in the network were intra-prokaryote associations (50.7% of a total of 231 links). In contrast, there were 30.7% phage–prokaryote links and 18.6% phage–phage links. Several *Enterobacteria* phages (T5, F1, 13a, RB14, RTP, and JSE) served as keystone nodes of the network, with a number of links to *Proteobacteria* species (Fig. 5). However, many of them belong to families *Comamonadaceae*, *Rhodocyclaceae*, and *Rhodobacteraceae*, rather than *Enterobacteria*, which might be attributed to the co-habitat of microbial members or broader host ranges of *Enterobacteria* phages. With exception of phage 13a, most *Enterobacteria* phages showed predominantly positive links to prokaryotic OTUs. In contrast, *Aeromonas* phage phiO18P, *Aeromonas* phage Aeh1, and *Mycobacterium* phage Che12 showed almost exclusively negative links to prokaryotic OTUs.

Strikingly, all of the phage–phage pairs except one were positively linked, suggesting a possibility of co-infection or co-habitat. Indeed, there were two independent modules exclusively comprised of phages (Fig. 5), which might represent core members. With the exception of *Pseudomonas* phage PP7, most of the core members were Caudovirales viruses, including *Enterobacteria* phage T5, *Morganella* phage MmP1, and *Burkholderia cepacia* complex phage BcepC6B.

### Sub-networks as functions of space and time

Four networks were constructed to explore the differences of phage–prokaryote interactions among anaerobic digesters (Additional file 1: Figure S4). The Ningbo-M network showed the largest average clustering coefficient and modularity than others but was the smallest network with the least numbers of nodes and modules (Additional file 1: Table S4). The Qingdao network showed the largest average path distance and size. In terms of phage composition, T7-like and T4-like phages were dominant in all networks. Other abundant phages included PhiC31-like phages (Ningbo-M) and N15-like phages (Qingdao).

To explore time dynamics of phage–prokaryote interaction, we generated four networks by dividing 12 months into four seasons (winter: October 2012–December 2012; spring: January 2013–March 2013; summer: April 2013–June 2013; and autumn: July 2013–September 2013) (Additional file 1: Figure S5). The winter network showed the highest *R* ^2^ of power-law, average path distance, and modularity (Additional file 1: Table S4). The spring network showed the largest average clustering coefficient, while the autumn network showed the largest average degree. Similar to space-based networks, T7-like and T4-like phages were prevalent in these networks. Other abundant phages included lambda-like phages (the winter network) and PhiC31-like phages (the summer network).

## Discussion

Bacteriophages are increasingly considered in efforts to describe factors controlling microbial productivity, community composition, and biogeochemical cycles [5, 20]. Phages are abundant in WWTPs since rich nutrients in activated sludge induce high microbial abundance and phage-to-prokaryote ratios [10, 20, 21], which cause significant phage-induced mortality to prokaryotic communities [22]. However, phage population dynamics and interaction with prokaryotic communities remain little understood. Total phage-like particles have been enumerated by epi-fluorescence microscopy [1] or FACS analysis [23], which provides little information regarding specific phage–host interactions. Although isolating phages and prokaryotes is powerful to address this task [21], it is challenging since the majority of phages are not readily cultivable under laboratory conditions. Based on DNA fingerprints, our study offers a glimpse into the population dynamics by demonstrating high variability in the spatiotemporal dynamics of phages and prokaryotes (Additional file 1: Figure S1 and S3).

Average dissimilarity of phage communities was 16.0% between two consecutive months (Additional file 1: Table S5). Substantial variations in relative abundance of different phage groups over time (Additional file 1: Figure S3) suggested that a large number of phages were produced and consumed in activated sludge, unveiling high activity of phages in sludge environment. Such dramatic fluctuation of phages, either temporally or spatially, may have important implications to ecosystem stability (e.g., the number of prokaryotic cells lysed), ecosystem function, and functional redundancy. A better understanding of the spatiotemporal extents of such fluctuation would be useful to determine phage production and decay rates. Interestingly, despite the high ephemeral variation of individual phages, a relatively stable, core phage community across space and time was notable (Additional file 1: Figure S2), which likely results in relatively steady and even predictable effects on prokaryotic communities and process parameters.

An important finding drawn from our data is the significant, positive correlation of α- and β-diversities between phage and prokaryotes (Fig. 1). As phages are obligate parasites of prokaryotes, their diversity is thus limited by the presence of their preys. If one assumes that each prokaryotic species is infected by at least one phage [24], phage diversity should be equal to prokaryotic diversity or even higher. However, it may be argued that phages detected in this study, relying on the design of phage probes on GeoChip, are likely gross underestimations. Because it is intractable to estimate the true size of the phage population, we assume that an equal proportion is missing for all samples, meaning that changes in α- and β-diversity detected by GeoChip reflect changes in total phage population. For the same reason that phages detected by GeoChip is a proportion of phage population, our calculation of phage contribution to 40.6% of total microbiome compositions (Table 1) is likely to be underestimated also. Therefore, phages may have a more central role in anaerobic digesters of WWTPs than previously expected, maintaining prokaryotic diversity necessary for functional performance of anaerobic digesters.

Both α- and β-diversity of phages and prokaryotes were positively correlated (Fig. 1), inferring that cell lysis could be a top-down control of prokaryotic communities as influenced by predator–prey dynamics. However, prokaryotic communities may also have shifted by changes in organic matter pools as a consequence of phage-mediated cell lysis, altering community structure from the bottom-up. Although we cannot distinguish between top-down and bottom-up controls, it is likely that top-down control may have more prominent influence on prokaryotic community in anaerobic digesters than natural environments such as ocean [2] and aquifer sediment [6], because microbiomes in anaerobic digesters are less likely to experience carbon and energy limitations that directly impact cell survival and maintenance.

Given the substantial influence of prokaryotic community composition by phages (Table 1), it is reasonable to believe that process parameters will be affected. Recently, phage lysis of prokaryotic cells was suggested to stimulate microbial functional groups to reduce cell-bound carbon in a bioreactor treating petroleum-refinery drainage water, resulting in higher than expected organic carbon removal [25]. In addition, decline in the phosphorus removal performance of a laboratory-scale wastewater treatment bioreactor was shown to associate with increases in phage-like particles capable of infecting *Accumulibacter*, the dominant phosphorus degrader [26]. Our PLS-PM results demonstrated that phages had strong effects on biogas production and volatile solid concentrations (Fig. 2), providing strong evidence that phage predation affected metabolic processes related to nutrient removal, organic matter mineralization, or other WWTP characteristics. For example, it was shown recently that lytic phages can be used to control overgrowth of filamentous bacteria *Sphaerotilus natans* and *Haliscomenobacter hydrossis*, raising the possibility to reduce sludge bulking in WWTPs [27]. Alternatively, the influence of phage on metabolic processes can be beneficial in that phage infection serves as an efficient strategy of horizontal gene transfer (transduction), which confers novel metabolic capacities to prokaryotic communities [28].

Associations between certain phages and prokaryotes have been documented by microbial association networks in marine systems [18, 29]. In this study, the majority of associations between and within prokaryote and phage pairs were only distantly related in phylogeny (Fig. 5), suggesting that synchronous oscillations of these prokaryotes and phages could be due to similar responses to environmental drivers rather than phage–host relationships. Alternatively, tight ecological coupling arising from auxotrophy, cross-feeding, competition, or predation could also lead to positive or negative correlations between and within prokaryote and phage pairs.

Each natural microbe is potentially targeted by a subset of the phage pool, resulting in positive or negative correlations between phage–prokaryote pairs [20]. Therefore, it is intriguing to note that the same phage at the genera level shows almost exclusively positive or negative links with prokaryotes (Fig. 5), suggesting that phage–prokaryote relationship is shallowly branched, such that the pairs are limited only within small, shallow viral clades. The ecological paradigms of a phage’s life strategy remain contentious, famously coined as kill-the-winner [30] and piggyback-the-winner models [31]. The switch between models is contingent on environmental nutrients, physiological conditions of the hosts, and phage types [32]. For example, *Myoviruses* favor kill-the-winner life strategy, but *Podoviruses* thrive in temperate-virus-enriched communities allowing for the piggyback-the-winner model.

Identification of phage–phage pairs can also be attributed to ecological connectedness of individual phages. The intraguild predators, which refer to two or more predator species within a given habitat, compete for the same prey, leading to negative interactions among predators [33]. The predominantly positive correlation among phages (Fig. 5) suggests that intraguild predation of prokaryotes by phages is scarce in anaerobic digesters of WWTPs. Rather, positive correlations in almost all of phage–phage pairs can be well explained by co-infection, since single bacterium can be susceptible to infection by multiple phages [34]. At least 26 discrete populations of double-stranded DNA phages from the same seawater sample were shown to infect a single *cyanobacterial* strain of *Synechococcus* [35]. Furthermore, nearly half of the 5492 microbial genomes with detectable phage signal contained more than one phage [36], demonstrating that phage breadth to infect the same host is grossly underestimated.

Most of the co-infections identified in the microbial genomes involved multiple *Caudovirales*. Consistently, we found that most of core members of phage modules were also *Caudovirales* viruses (Fig. 5). Although most phages display very narrow host specificities [37], there has been evidence showing that phages isolated from WWTPs have unusually broad host ranges, with some capable of lysing both Gram-negative and Gram-positive bacteria [38]. Those polyvalent phages are of particular interests for potential applications as a convenient antibacterial tool. However, we need to admit that a number of phage–phage pairs discovered in this study can result from infection of individual hosts sharing similar habitats.

## Conclusions

We show here that phages affect prokaryotic community diversity and composition in anaerobic digesters of WWTPs, which represent a first attempt to simultaneously track phage and prokaryotic dynamics across time and space in an engineered system. Frequent fluctuations in both phages and prokaryotes are observed throughout the study period, demonstrating stable microbiome composition which is not likely to evolve. This contrasts to the observation that anaerobic digesters appear to be in a steady state since they are carefully maintained in the full-scale WWTPs, suggesting that steady processes are amenable and derivable from assumedly stochastic behavior of individual phages and prokaryotes. Our results indicate that phage and prokaryotic communities are highly interconnected, unveiling keystone phage–prokaryote pairs that might sustain ecosystem stability and function over time. In particular, the observation of two modules exclusively composed of phages warrants attention to understand their implication on prokaryotic communities and ecological processes. In moving forward, understanding phage dynamics and interaction with prokaryotic community requires a critical examination of their associations at the functionally relevant spatiotemporal scale, which help unveil a phage’s impact on process performance. Therefore, this study provides much needed information for basic science of phage–prokaryote interaction and practical application to improve process performance.

## Methods

### Collection of anaerobic digester samples

We collected samples from anaerobic digesters in three full-scale WWTPs located in Beijing of northern China, Qingdao, Shandong Province of mid-China, and Ningbo, Zhejiang Province of southern China. WWTPs of Beijing and Qingdao use mesophilic (35–38 °C) anaerobic digesters to treat activated sludge, while the WWTP of Ningbo uses a two-phase anaerobic technique with both mesophilic (40 °C) and thermophilic (45 °C) anaerobic digesters. The Beijing WWTP treats mainly excess sewage sludge, the Qingdao WWTP treats mainly municipal solid waste, and the Ningbo WWTP treats excess sewage sludge and livestock manure. All the WWTPs were maintained in good condition during our study period, with methane steadily comprising of 60–70% of generated biogas.

Samples were collected on the monthly basis from October, 2012 to September, 2013. Briefly, 2 l of each sludge sample were obtained near the outlets of anaerobic digester tanks, stored in a portable ice container and immediately transported to the laboratory. A large part of each sample was kept at 4 °C to analyze physicochemical properties, while the rest was precipitated by centrifugation at 14,000*g* for 10 min. After centrifugation, the pellet was air-dried for 30 min and stored at −80 °C for DNA extraction, while supernatant was decanted. It was noted that solid contents of all samples were less than 10%.

### Measurements of physicochemical properties

Measurements of trace metals containing Fe, Cu, and Cr and inorganic anion containing sulfate (SO_4_^2-^), chloride (Cl^-^), and nitrogen (NH_4_^+^-N) concentrations were based on standard protocols [39], using an Inductive Coupled Plasma Emission Spectrometer (ICP) (PerkinElmer, Waltham, MA, USA). The organic loading rate of each sludge sample, including total solid (TS), volatile solid (VS), and chemical oxygen demand (COD), were also measured using standard protocols [39]. pH was determined by a Hach HQ40d meter (Hach, Loveland, CO, USA). Biogas volume produced in the Qingdao anaerobic tank was monitored daily by a high-precision wet-type flow gas meter TG-01-Series (Ritter, Bochum, Ruhr, Germany).

### DNA extraction and experiments with MiSeq sequencing and GeoChip

DNA was extracted by the PowerSoil DNA isolation kit (MoBio Laboratories, Carlsbad, CA, USA) and then purified by agarose gel electrophoresis followed by extractions with phenol and chloroform and precipitation with butanol. Subsequently, a ND-1000 spectrophotometer (NanoDrop Technologies, Wilmington, DE, USA) was used to assess DNA quality, confirming that the A_260_/A_280_ ratio was between 1.8 and 2.0 and A_260_/A_230_ over 1.5.

We used a MiSeq equipment (Illumina, San Diego, CA, USA) to sequence the microbiome composition of sludge samples. The primer sets 515F (5′-GTG CCA GCM GCC GCG GTA A-3′) and 806R (5′-GGA CTA CHV GGG TWT CTA AT-3′) were used to amplify the V4 region of 16S rRNA gene, which provides vast taxonomic prokaryotic groups [40]. Triplicates of amplification were conducted for each sample before PCR amplification under thermal cycling condition [41]. Subsequently, we purified PCR products through QIAquick Gel Extraction Kit (Qiagen, Valencia, CA, USA) and established sample libraries according to the manufacturer’s manual. Quality trimming was conducted with our Galaxy pipeline. Poor quality sequences (too short or obscure sequences) were removed, resulting in a total of 56,143 sequences in all of the 48 samples.

GeoChip 5.0 M, the newest version of functional gene array containing 455 phage probes, was used. For each sludge sample, 1 μg of purified DNA was labeled with the fluorescent dye Cy-3 (GE Healthcare, Little Chalfont, UK) using random priming as described previously [42], then purified by a QIAquick Purification kit (Qiagen, Valencia, CA, USA), and dried at 45 °C for 45 min by a SpeedVac equipment (Thermo Savant, NY, USA). DNA was then resuspended in 27.5 μl of DNase/RNase-free distilled water and evenly mixed with 99.4 μl of hybridization solution, which contained final concentrations of 1× Acgh blocking, 1× HI-RPM hybridization buffer, 10 pM universal standard DNA, 0.05 μg/μl Cot-1 DNA, and 10% formamide. Mixed solution was kept at 90 °C for 3 min for denaturation and incubated at 37 °C for 30 min before carrying out GeoChip hybridization at 67 °C for 24 h with a rotation at 20 rpm in a G2545A hybridization oven (Agilent, Santa Clara, CA, USA). After removing unbounded DNA at room temperature, GeoChip was scanned with a MS200 Microarray Scanner (Roche NimbleGen, Madison, WI, USA) at 633 nm. Images were quantified by Feature Extraction software 11.5.1.1 (Agilent, Santa Clara, CA, USA). The raw microarray data was processed by GeoChip Microarray Data Manager pipeline (http://ieg.ou.edu/microarray/) as previously described [43], using the following steps: (i) remove the spots with a signal-to-noise ratio (SNR) less than 2.0, (ii) log-transformed the data and then, on each microarray, divide them by the mean intensity of all the genes, and (iii) remove genes detected only once in each digester.

### Data analyses

The richness index was based on the number of prokaryotic OTUs or phage genes detected in each sample. The α-diversity was represented as Shannon’s index, and the β-diversity was based on Bray-Curtis distance. Detrended correspondence analysis (DCA) was performed to illustrate the spatiotemporal dynamics of phages and prokaryotes. Mrpp (multi-response permutation procedure), Anosim (analysis of similarities), and Adonis (permutational multivariate analysis of variance) based on Bray-Curtis distance were used to examine the differences in community composition. Multiple regression matrix (MRM) and partial least squares path modeling (PLS-PM) were carried out to explore relationships among phages, prokaryotes, and process parameters, using functions in the Vegan (v. 2.4-1), Ecodist (v. 1.2.9), and Plspm (v. 0.4.7) packages in R (v. 3.1.1) (http://www.r-project.org).

Association networks were constructed with an online Molecular Ecological Network Analyses Pipeline (MENAP) (http://ieg2.ou.edu/MENA) as described previously [19, 44]. Since the numbers of prokaryotic OTUs and phages varied considerably by samples, only prokaryotic OTUs and phages detected in at least 75% of all samples were used. Similarity matrices were calculated based on Spearman rank correlation. Thresholds of networks were determined by a random matrix theory (RMT)-based algorithm, and modules were separated based on greedy modularity optimization [45]. Cytoscape 3.2.1 was used to depict the networks, showing nodes (individual prokaryotic OTUs or phages) linked by lines that denoted positive or negative correlations.

## Additional file

Additional file 1: Figure S1. Spatiotemporal changes in (A) richness of phages, (B) richness of prokaryotes, (C) the α-diversity of phages and (D) the α-diversity of prokaryotes. *BJ* Beijing samples, *QD* Qingdao samples, *Ningbo-M*: samples from Ningbo anaerobic digester maintained at mesophilic temperature, and *Ningbo-T* samples from Ningbo anaerobic digester maintained at thermophilic temperature. **Figure S2.** Proportions of phages based on their detection frequencies in anaerobic digesters. **Figure S3.** The most abundant phages detected in Beijing samples as functions of (A) time and their average relative abundance in samples across (B) space. **Figure S4.** Association networks generated from (A) Beijing, (B) Ningbo-T, (C) Ningbo-M, and (D) Qingdao samples. Modules with equal or less than five nodes were omitted. Positive linkages are shown in red edges, while negative linkages are shown in blue edges. Spearman’s correlation coefficients are indicated by line width. **Figure S5.** Association networks generated according to seasons: (A) winter, (B) spring, (C) summer, and (D) autumn. Modules with equal or less than five nodes were omitted. Positive linkages are shown in red edges, while negative linkages are shown in blue edges. Spearman’s correlation coefficients are indicated by line width. **Table S1.** Dissimilarity tests of Mrpp, Anosim, and Adonis on community structures. **Table S2.** Measurements of physicochemical properties related to process performance. **Table S3.** Taxonomic information of *Euryarchaeota* OTUs. **Table S4.** Major properties of association networks. **Table S5.** Dissimilarity of phage communities between months represented as β-diversity. Boldface values indicate dissimilarities between two consecutive months. (DOCX 5058 kb)

## Abbreviations

MRM: Multiple regression matrix
PLS-PM: Partial least squares path model
VFA: Volatile fatty acids
WWTPs: Wastewater treatment plants
