# Supplementary material for: Bacteriophage–prokaryote dynamics and interaction within anaerobic digestion processes across time and space
Source: Microbiome. 2017 May 31;5:57. doi: 10.1186/s40168-017-0272-8 (PMC5452288; doi:10.1186/s40168-017-0272-8)
Supplement: Additional file 1: Figure S1. — Spatiotemporal changes in (A) richness of phages, (B) richness of prokaryotes, (C) the α-diversity of phages and (D) the α-diversity of prokaryotes. BJ Beijing samples, QD Qingdao samples, Ningbo-M: samples from Ningbo anaerobic digester maintained at mesophilic temperature, and Ningbo-T samples from Ningbo anaerobic digester maintained at thermophilic temperature. Figure S2. Proportions of phages based on their detection frequencies in anaerobic digesters. Figure S3. The most abundant phages detected in Beijing samples as functions of (A) time and their average relative abundance in samples across (B) space. Figure S4. Association networks generated from (A) Beijing, (B) Ningbo-T, (C) Ningbo-M, and (D) Qingdao samples. Modules with equal or less than five nodes were omitted. Positive linkages are shown in red edges, while negative linkages are shown in blue edges. Spearman’s correlation coefficients are indicated by line width. Figure S5. Association networks generated according to seasons: (A) winter, (B) spring, (C) summer, and (D) autumn. Modules with equal or less than five nodes were omitted. Positive linkages are shown in red edges, while negative linkages are shown in blue edges. Spearman’s correlation coefficients are indicated by line width. Table S1. Dissimilarity tests of Mrpp, Anosim, and Adonis on community structures. Table S2. Measurements of physicochemical properties related to process performance. Table S3. Taxonomic information of Euryarchaeota OTUs. Table S4. Major properties of association networks. Table S5. Dissimilarity of phage communities between months represented as β-diversity. Boldface values indicate dissimilarities between two consecutive months. (DOCX 5058 kb) [file 40168_2017_272_MOESM1_ESM.docx]

**
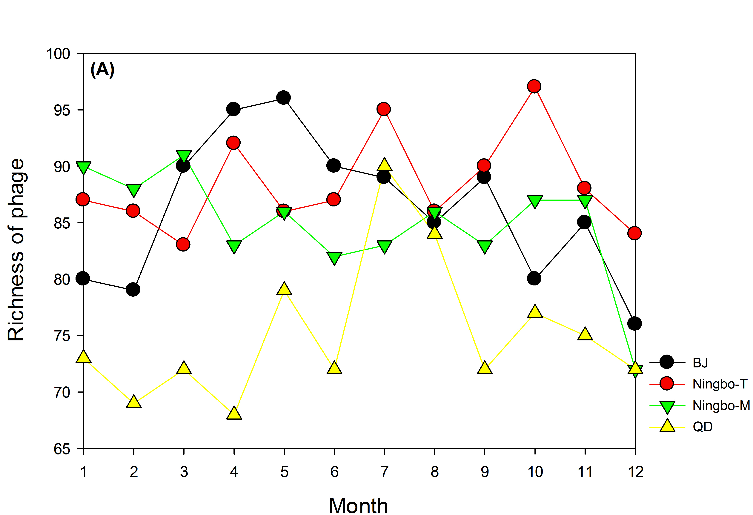

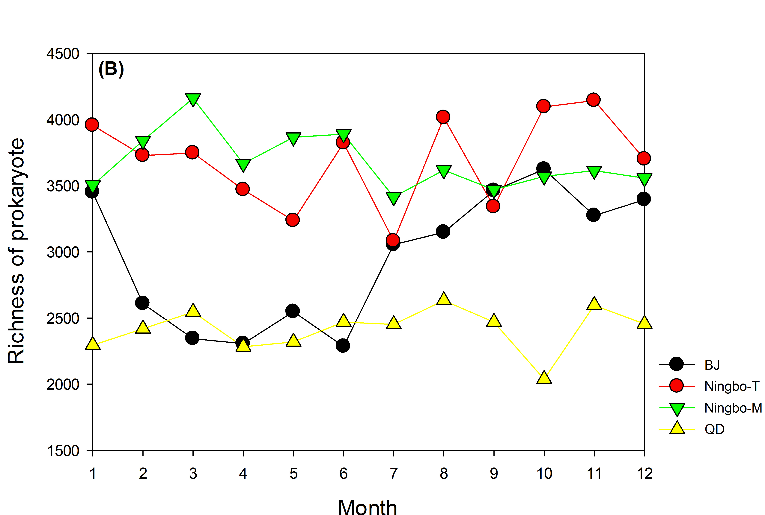
**

**
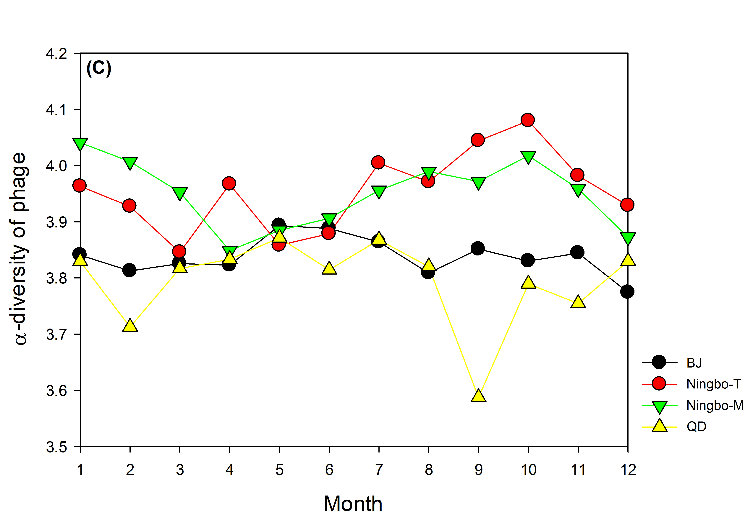

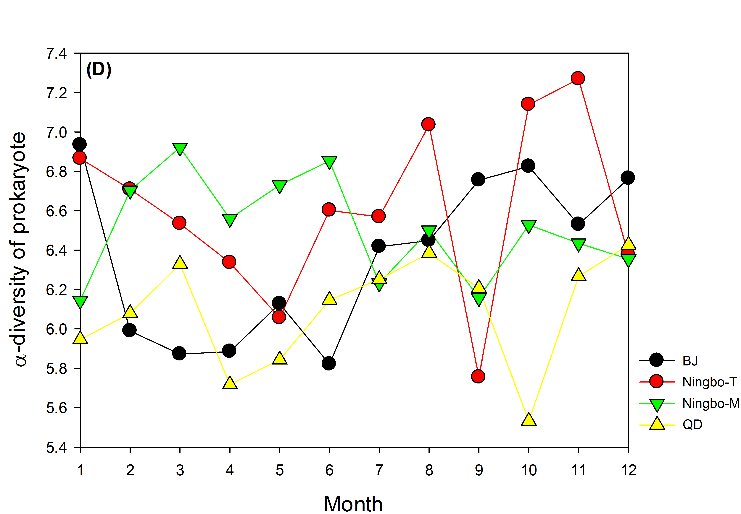
**

**Fig. S1.** Spatiotemporal changes in (A) richness of phages, (B) richness of prokaryotes, (C) the α-diversity of phages and (D) the α-diversity of prokaryotes. BJ: Beijing samples; QD: Qingdao samples; Ningbo-M: samples from Ningbo anaerobic digester maintained at mesophilic temperature, and Ningbo-T: samples from Ningbo anaerobic digester maintained at thermophilic temperature.


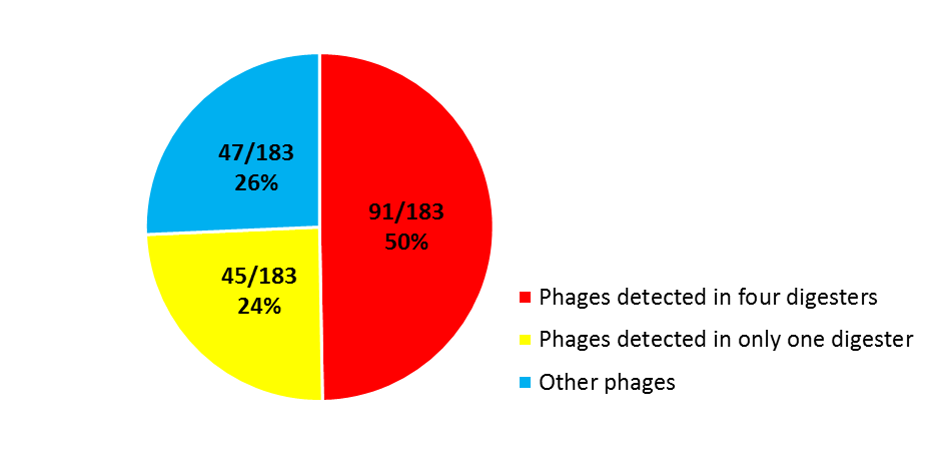


**Fig. S2.** Proportions of phages based on their detection frequencies in anaerobic digesters.

**
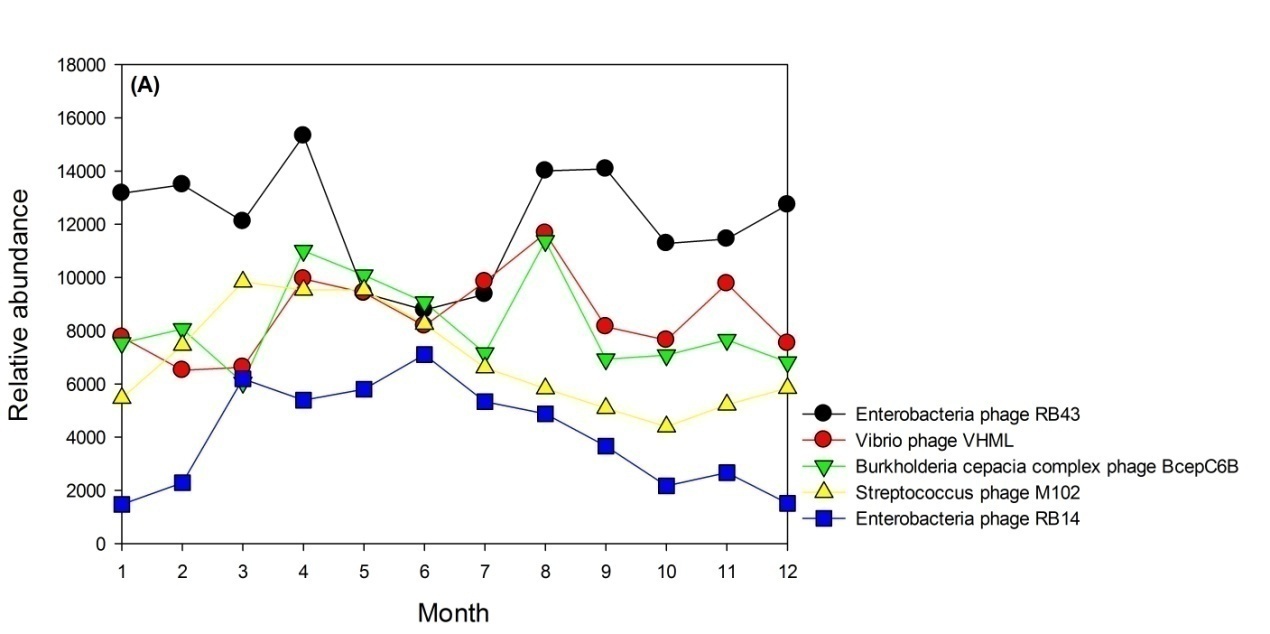
**


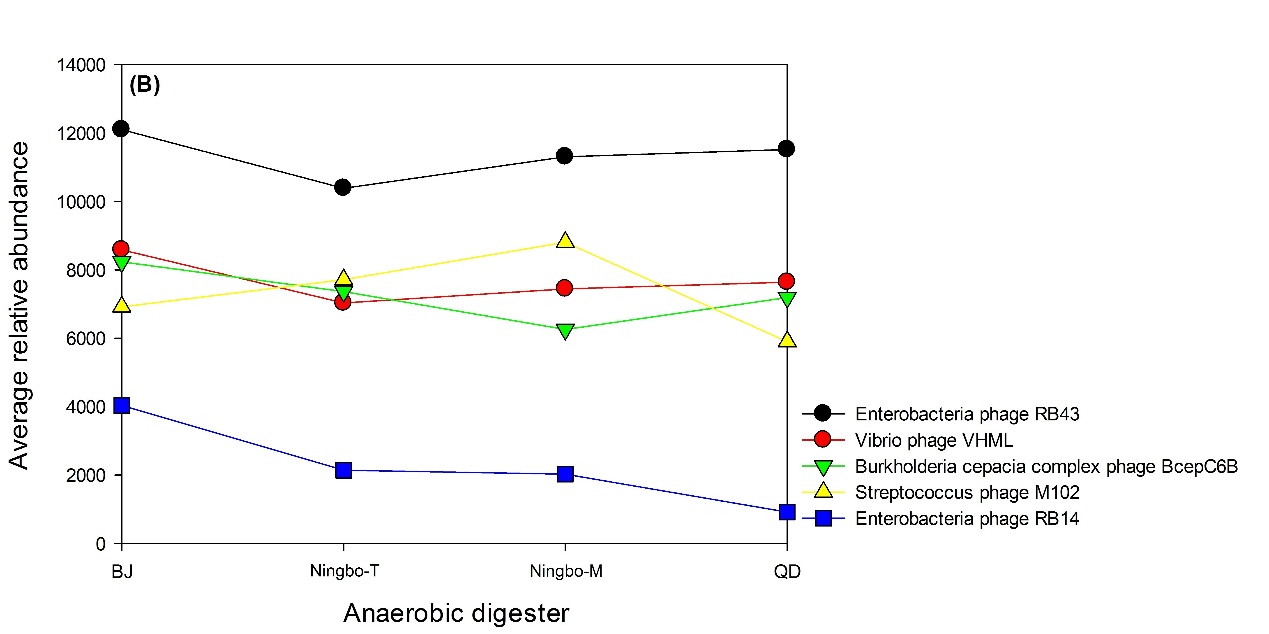


**Fig. S3.** The most abundant phages detected in Beijing samples as functions of (A) time and their average relative abundance in samples across (B) space.


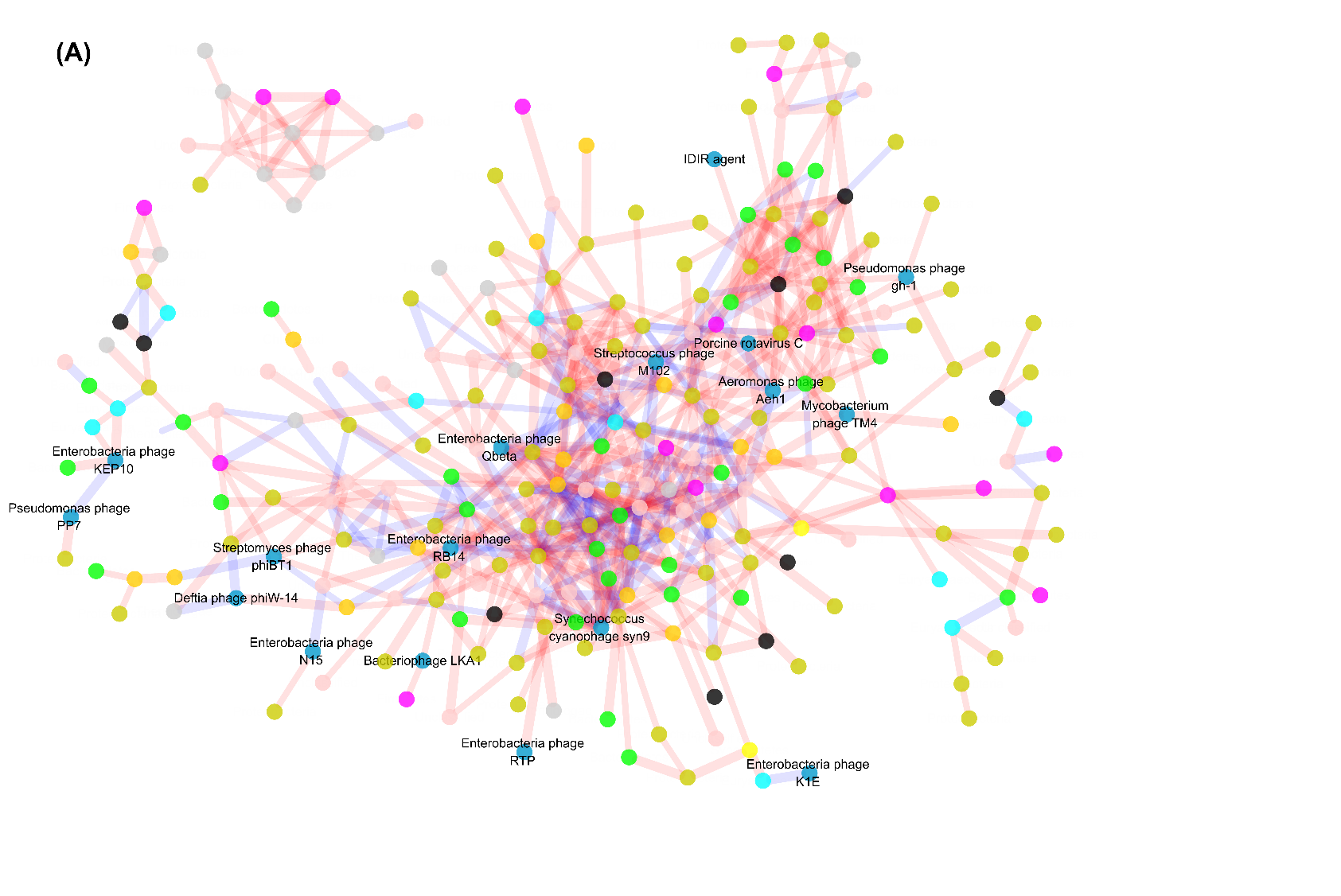


**
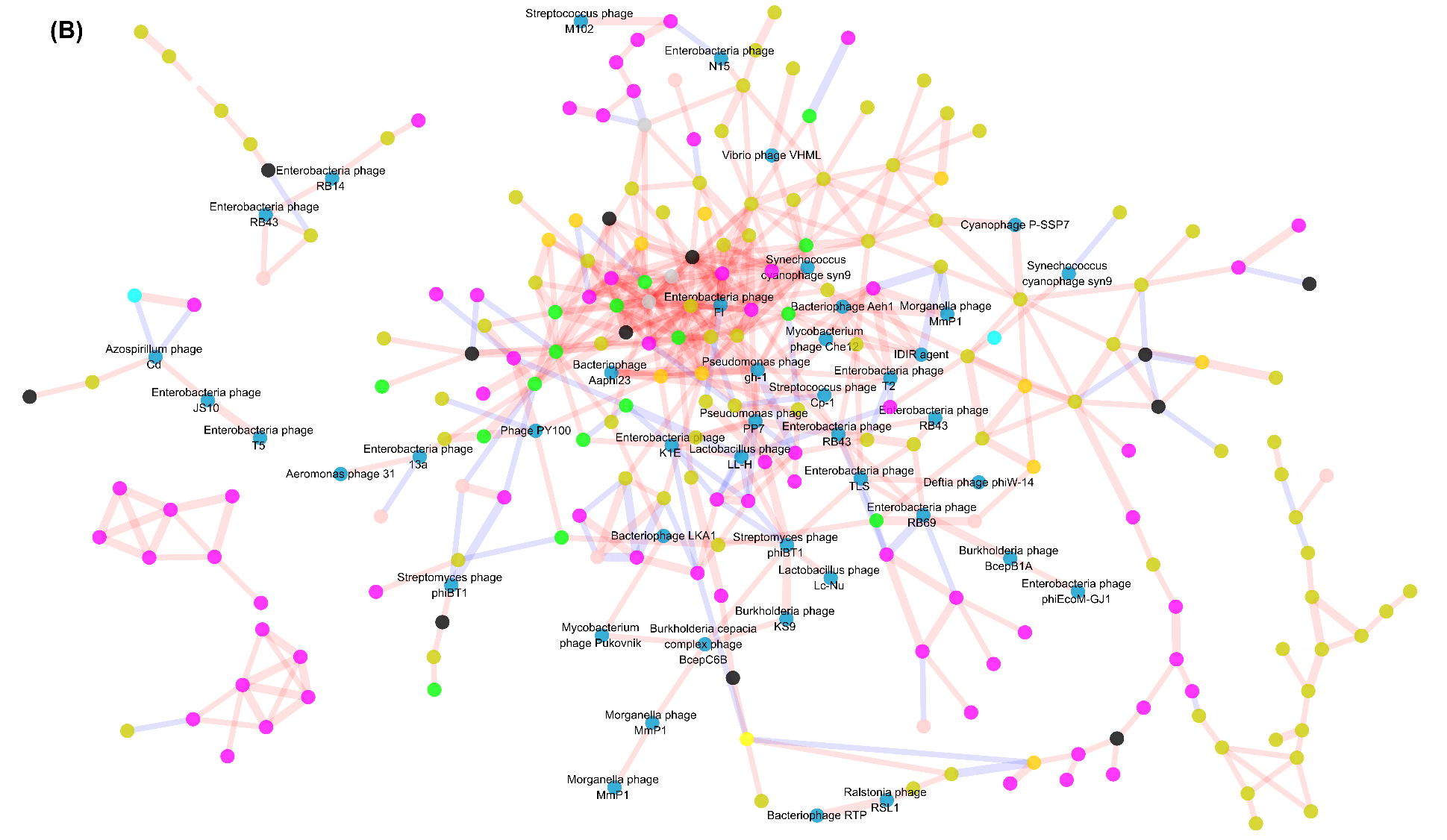
**

**
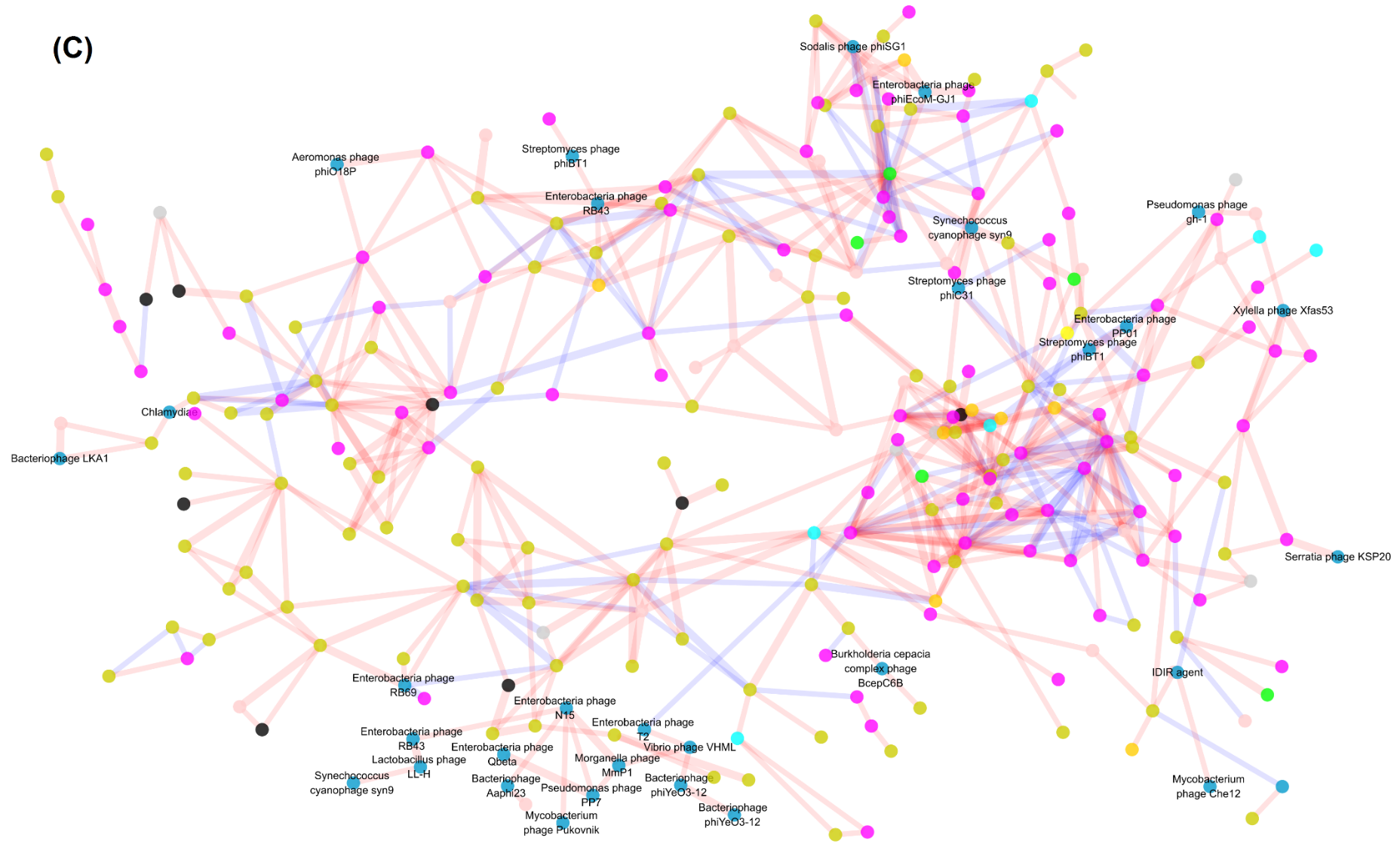
**

**
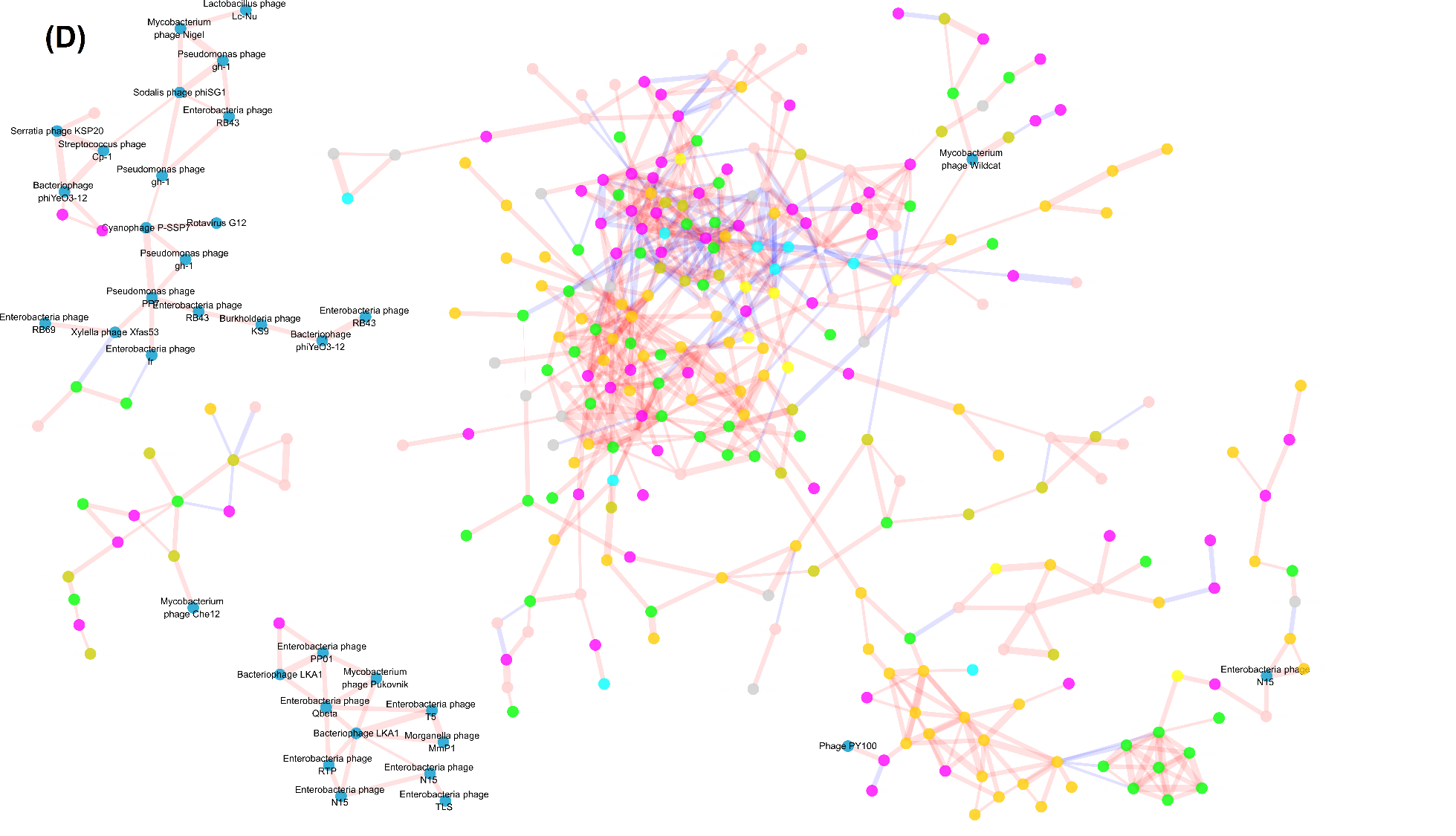
**

**Fig. S4.** Association networks generated from (A) Beijing, (B) Ningbo-T, (C) Ningbo-M and (D) Qingdao samples. Modules with equal or less than 5 nodes were omitted. Positive linkages are shown in red edges, while negative linkages are shown in blue edges. Spearman’s correlation coefficients are indicated by line width.


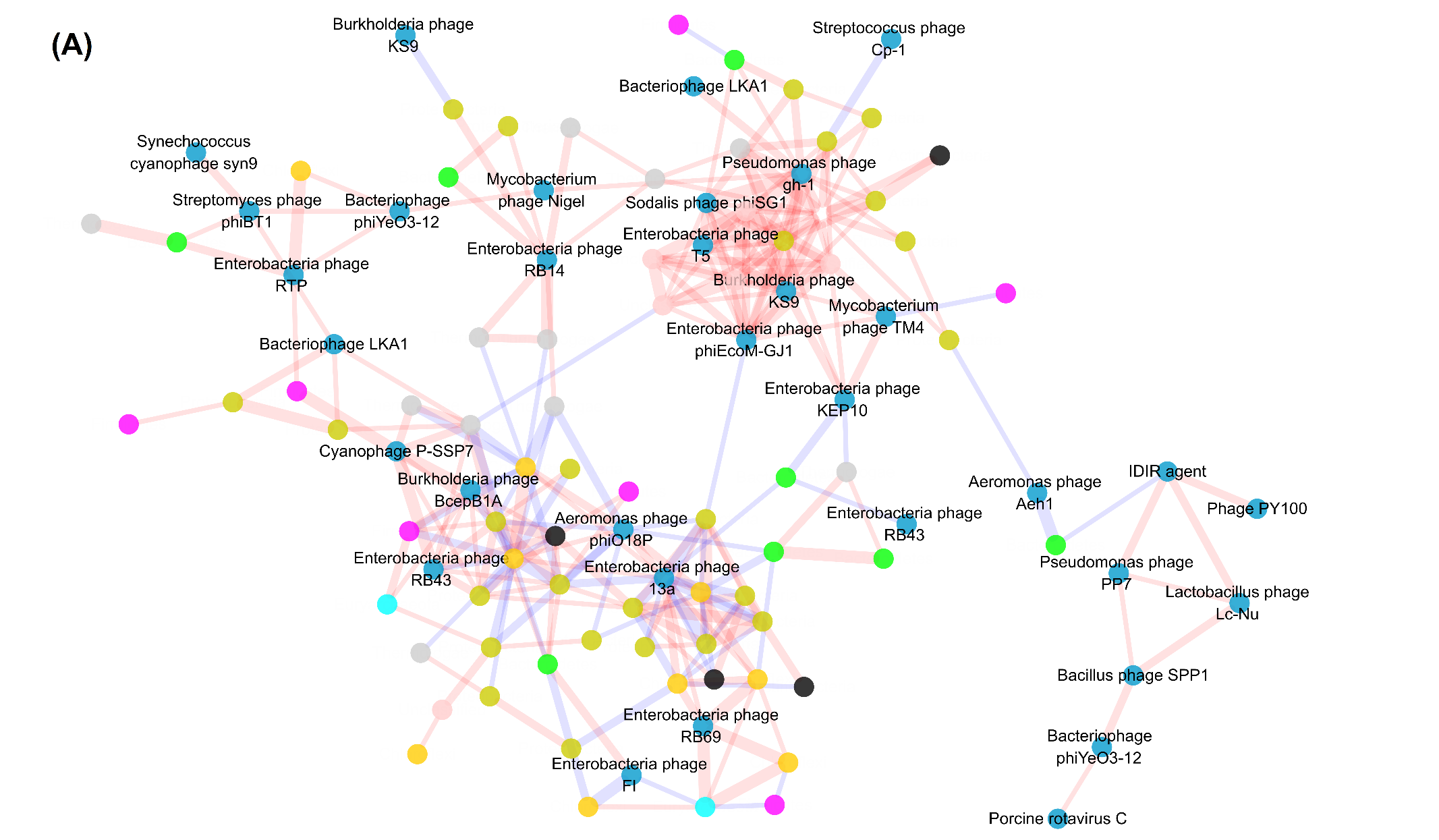


**
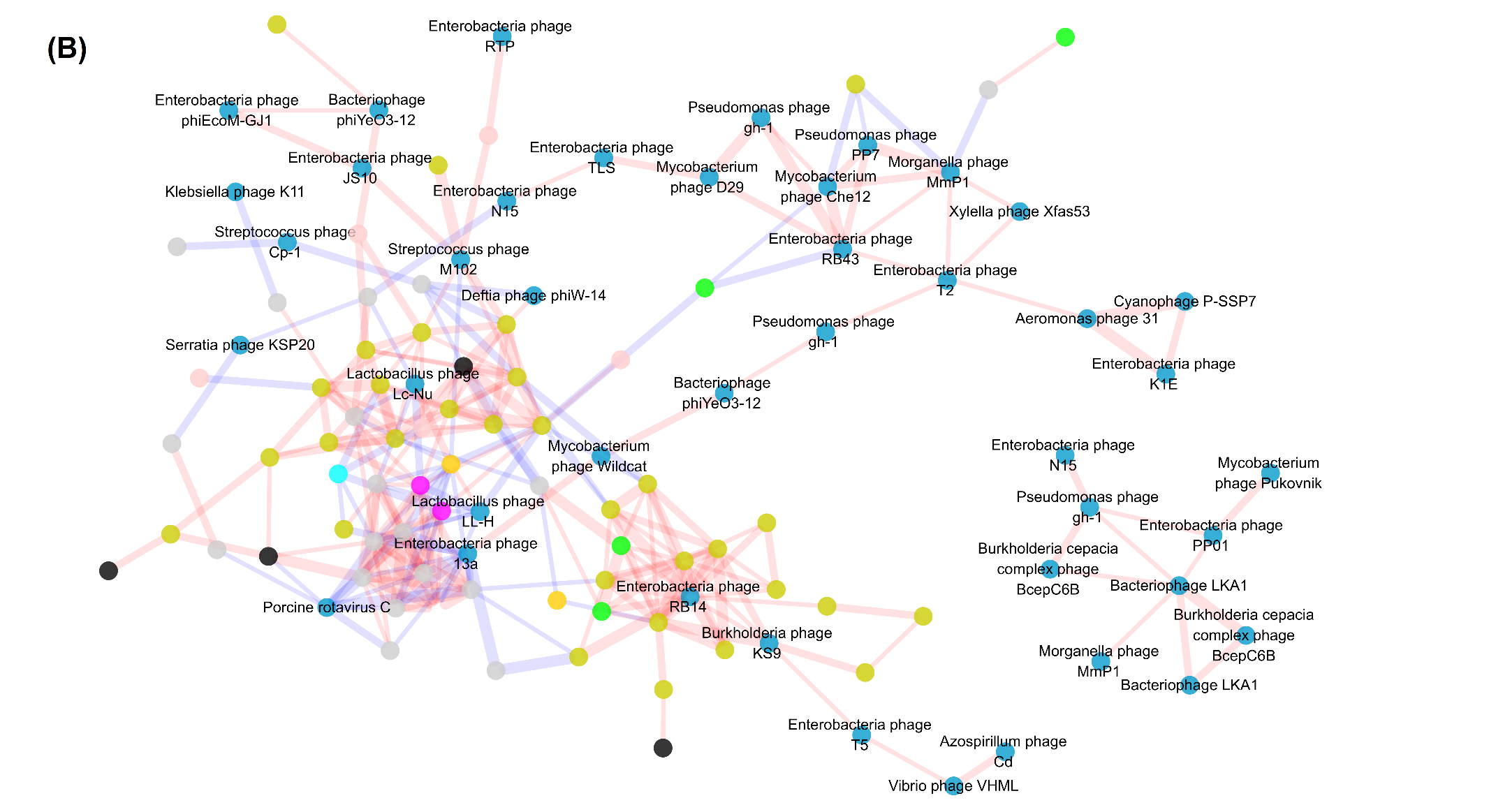
**


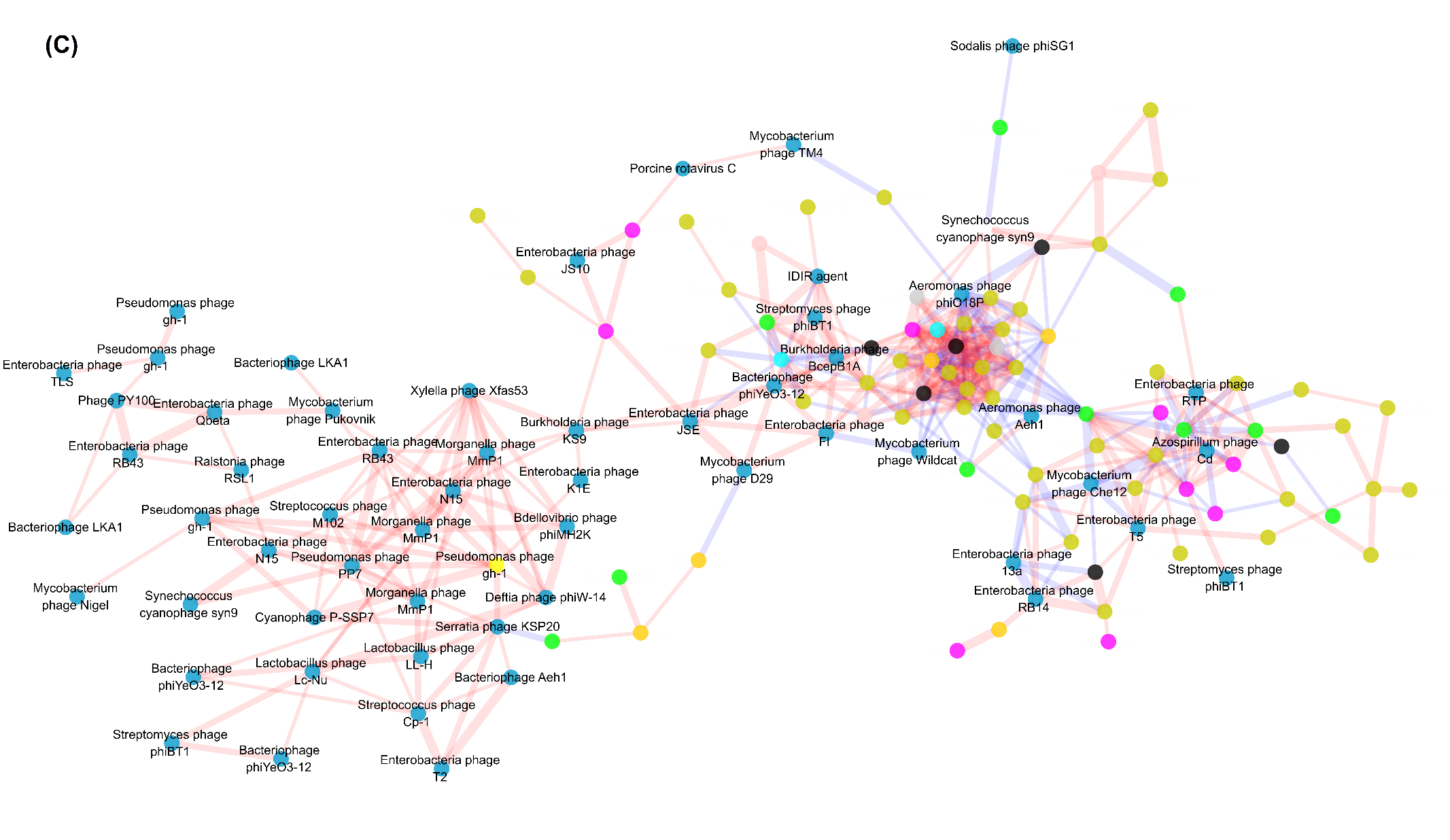


**
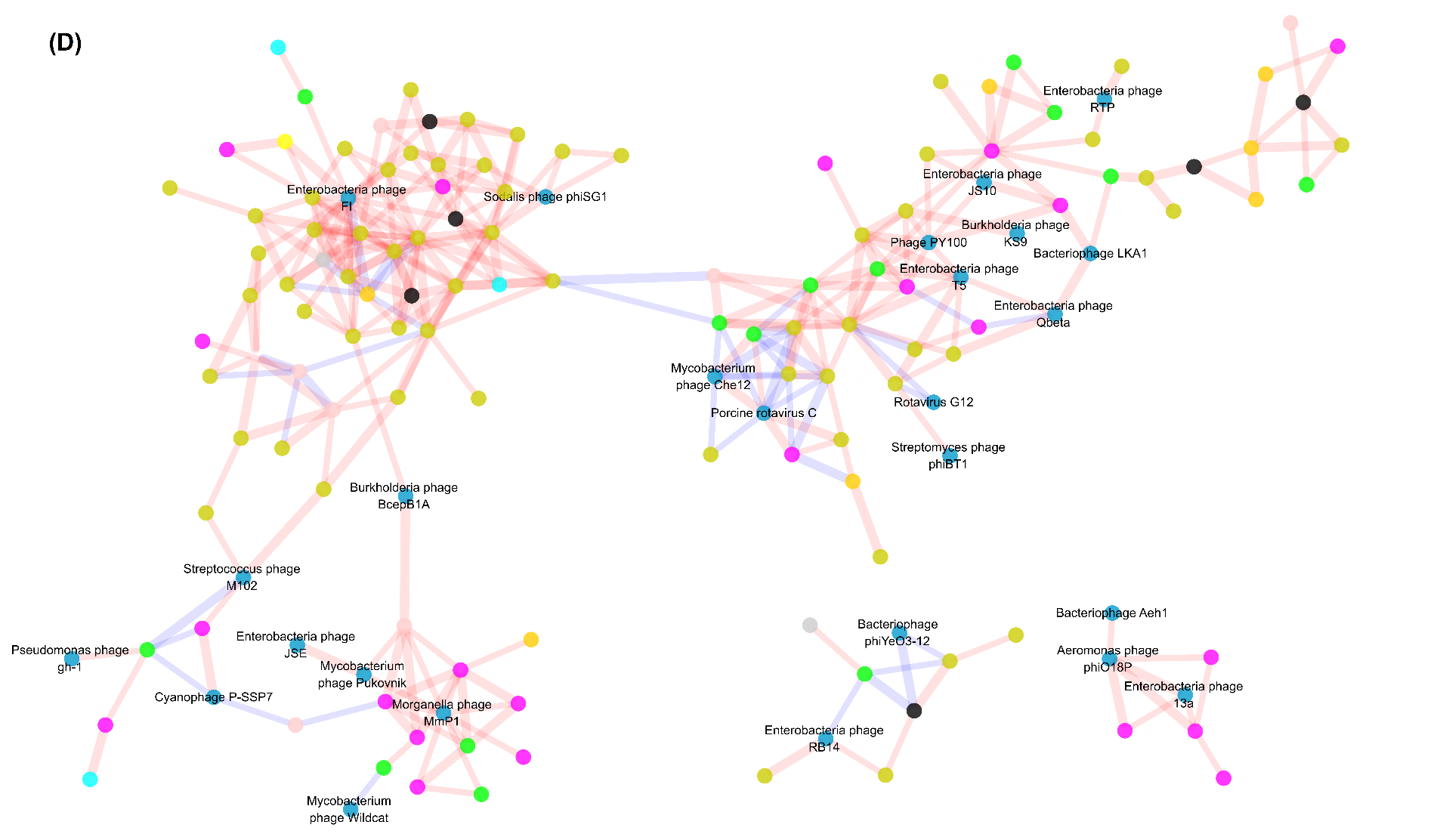
**

**Fig. S5.** Association networks generated according to seasons: (A) Winter, (B) Spring, (C) Summer and (D) Autumn. Modules with equal or less than 5 nodes were omitted. Positive linkages are shown in red edges, while negative linkages are shown in blue edges. Spearman’s correlation coefficients are indicated by line width.

**Table S1.** Dissimilarity tests of Mrpp, Anosim and Adonis on community structures.

| Groups | Mrpp^a^ | | Anosim | | Adonis | |
| --- | --- | --- | --- | --- | --- | --- |
|  | δ | P-value | Statistic R | P-value | Statistic R^2^ | P-value |
| BJ vs Ningbo-T | 0.183 | **0.002^b^** | 0.254 | **0.004** | 0.142 | **0.001** |
| BJ vs Ningbo-M | 0.163 | **<0.001** | 0.512 | **0.001** | 0.217 | **0.002** |
| BJ vs QD | 0.207 | **<0.001** | 0.417 | **0.001** | 0.236 | **0.001** |
| Ningbo-T vs Ningbo-M | 0.167 | **0.024** | 0.19 | **0.005** | 0.075 | 0.060 |
| Ningbo-T vs QD | 0.222 | **0.001** | 0.353 | **0.002** | 0.2 | **0.001** |
| Ningbo-M vs QD | 0.2 | **0.001** | 0.423 | **0.001** | 0.225 | **0.001** |

^a^Abbreviations: Mrpp, multi-response permutation procedure; Anosim, analysis of similarity; Adonis, non-parametric multivariate analysis of variance with the Adonis function.

^b^Boldface values indicate significant differences (P<0.050).

**Table S2.** Measurements of physicochemical properties related to process performance.

| Sample | TS^a^ (mg/mL) | VS (mg/mL) | Gas (m^3^/d) | COD (mg/L) |
| --- | --- | --- | --- | --- |
| BJ1 | 14.26 | 8.44 | - | 24.26 |
| BJ2 | 17.83 | 9.67 | - | 62.18 |
| BJ3 | 23.47 | 14.35 | - | 199.12 |
| BJ4 | 14.04 | 8.60 | - | 28.41 |
| BJ5 | 17.90 | 10.67 | - | 247.31 |
| BJ6 | 5.94 | 2.80 | - | 52.63 |
| BJ7 | 20.53 | 10.01 | - | 115.79 |
| BJ8 | 29.52 | 12.65 | - | 9.43 |
| BJ9 | 15.44 | 25.08 | - | 56.34 |
| BJ10 | 15.19 | 8.86 | - | 18.00 |
| BJ11 | 3.51 | 2.01 | - | 30.00 |
| BJ12 | 12.25 | 7.73 | - | 36.00 |
| Ningbo-M1 | 84.15 | 34.79 | - | 246.63 |
| Ningbo-M2 | 65.43 | 26.15 | - | 99.15 |
| Ningbo-M3 | 60.15 | 24.12 | - | 78.72 |
| Ningbo-M4 | 84.16 | 34.80 | - | 107.49 |
| Ningbo-M5 | 68.38 | 27.57 | - | 197.36 |
| Ningbo-M6 | 58.44 | 26.56 | - | 62.50 |
| Ningbo-M7 | 79.72 | 32.57 | - | 395.70 |
| Ningbo-M8 | 61.72 | 24.15 | - | 84.21 |
| Ningbo-M9 | 98.96 | 36.71 | - | 136.84 |
| Ningbo-M10 | 94.27 | 31.31 | - | 89.62 |
| Ningbo-M11 | 122.09 | 42.87 | - | 169.70 |
| Ningbo-M12 | 26.07 | 22.45 | - | 81.00 |
| QD1 | 12.51 | 4.84 | 8692.97 | 70.64 |
| QD2 | 12.51 | 4.84 | 10116.07 | 82.13 |
| QD3 | 14.47 | 5.38 | 13419.16 | 131.06 |
| QD4 | 21.26 | 4.22 | 12573.21 | 101.42 |
| QD5 | 21.50 | 7.39 | 15376.43 | 116.30 |
| QD6 | 19.23 | 7.59 | 13698.46 | 103.41 |
| QD7 | 13.02 | 4.34 | 10664.73 | 298.92 |
| QD8 | 10.53 | 3.18 | 7818.60 | 84.21 |
| QD9 | 17.18 | 5.27 | 7740.30 | 31.58 |
| QD10 | 13.11 | 3.97 | 7238.42 | 42.45 |
| QD11 | 14.04 | 5.63 | 7665.04 | 107.04 |
| QD12 | 12.53 | 5.12 | 7577.07 | 66.00 |

^a^Abbreviations: BJ: Beijing; QD: Qingdao; TS, total solid content; VS, volatile solid content; Gas, average daily biogas production; COD, chemical oxygen demand; NH_3_-N, ammonia nitrogen.

**Table S3.** Taxonomic information of *Euryarchaeota* OTUs.

| OTU | Kingdom | Phylum | Class | Order | Family | Genus |
| --- | --- | --- | --- | --- | --- | --- |
| OTU_1019 | Archaea | Euryarchaeota | Methanobacteria | Methanobacteriales | Methanobacteriaceae | Methanobacterium |
| OTU_107 | Archaea | Euryarchaeota | Methanobacteria | Methanobacteriales | Methanobacteriaceae | Methanobacterium |
| OTU_1073 | Archaea | Euryarchaeota | Methanomicrobia | Methanomicrobiales | Methanomicrobiales-  _incertae_sedis | Methanolinea |
| OTU_1087 | Archaea | Euryarchaeota | Methanobacteria | Methanobacteriales | Methanobacteriaceae | Methanobacterium |
| OTU_1104 | Archaea | Euryarchaeota | Methanomicrobia | Methanomicrobiales | Methanomicrobiales-  _incertae_sedis | Methanolinea |
| OTU_1106 | Archaea | Euryarchaeota | Methanomicrobia | Methanomicrobiales | Methanomicrobiales-  _incertae_sedis | Methanolinea |
| OTU_1109 | Archaea | Euryarchaeota | Methanomicrobia | Methanosarcinales | Methanosaetaceae | Methanosaeta |
| OTU_1112 | Archaea | Euryarchaeota | Methanomicrobia | Methanomicrobiales | Methanomicrobiales-  _incertae_sedis | Methanoregula |
| OTU_1141 | Archaea | Euryarchaeota | Methanomicrobia | Methanosarcinales | Methanosaetaceae | Methanosaeta |
| OTU_115639 | Archaea | Euryarchaeota | Methanomicrobia | Methanomicrobiales | Methanomicrobiales-  _incertae_sedis | Methanolinea |
| OTU_1162 | Archaea | Euryarchaeota | Methanomicrobia | Methanomicrobiales | Methanomicrobiales-  _incertae_sedis | Methanolinea |
| OTU_1169 | Archaea | Euryarchaeota | Methanobacteria | Methanobacteriales | Methanobacteriaceae | Methanobacterium |
| OTU_1174 | Archaea | Euryarchaeota | Methanomicrobia | Methanomicrobiales | Methanomicrobiaceae | Methanosphaerula |
| OTU_1177 | Archaea | Euryarchaeota | Methanomicrobia | Methanomicrobiales | Methanomicrobiales-  _incertae_sedis | Methanolinea |
| OTU_1179 | Archaea | Euryarchaeota | Methanomicrobia | Methanomicrobiales | Methanomicrobiales-  _incertae_sedis | Methanolinea |
| OTU_118 | Archaea | Euryarchaeota | Methanobacteria | Methanobacteriales | Methanobacteriaceae | Methanobacterium |
| OTU_1185 | Archaea | Euryarchaeota | Methanomicrobia | Methanosarcinales | Methanosaetaceae | Methanosaeta |
| OTU_1205 | Archaea | Euryarchaeota | Methanomicrobia | Methanomicrobiales | Methanospirillaceae | Methanospirillum |
| OTU_1210 | Archaea | Euryarchaeota | Methanomicrobia | Methanomicrobiales | Methanomicrobiales-  _incertae_sedis | Methanolinea |
| OTU_1215 | Archaea | Euryarchaeota | Methanomicrobia | Methanomicrobiales | Methanospirillaceae | Methanospirillum |
| OTU_1274 | Archaea | Euryarchaeota | Methanomicrobia | Methanomicrobiales | Methanospirillaceae | Methanospirillum |
| OTU_1276 | Archaea | Euryarchaeota | Methanobacteria | Methanobacteriales | Methanobacteriaceae | Methanobacterium |
| OTU_12843 | Archaea | Euryarchaeota | Methanomicrobia | Methanomicrobiales | Methanomicrobiales-  _incertae_sedis | Methanolinea |
| OTU_1319 | Archaea | Euryarchaeota | Methanomicrobia | Methanosarcinales | Methanosaetaceae | Methanosaeta |
| OTU_1335 | Archaea | Euryarchaeota | Methanomicrobia | Methanomicrobiales | Methanomicrobiale-  s_incertae_sedis | Methanolinea |
| OTU_1351 | Archaea | Euryarchaeota | Methanomicrobia | Methanomicrobiales | Methanospirillaceae | Methanospirillum |
| OTU_1358 | Archaea | Euryarchaeota | Methanomicrobia | Methanomicrobiales | Methanospirillaceae | Methanospirillum |
| OTU_1359 | Archaea | Euryarchaeota | Methanomicrobia | Methanomicrobiales | Methanospirillaceae | Methanospirillum |
| OTU_1391 | Archaea | Euryarchaeota | Methanobacteria | Methanobacteriales | Methanobacteriaceae | Methanobacterium |
| OTU_1393 | Archaea | Euryarchaeota | Methanomicrobia | Methanomicrobiales | Methanomicrobiales-  _incertae_sedis | Methanolinea |
| OTU_1409 | Archaea | Euryarchaeota | Methanomicrobia | Methanomicrobiales | Methanospirillaceae | Methanospirillum |
| OTU_1443 | Archaea | Euryarchaeota | Methanomicrobia | Methanomicrobiales | Methanomicrobiaceae | Methanosphaerula |
| OTU_1456 | Archaea | Euryarchaeota | Methanobacteria | Methanobacteriales | Methanobacteriaceae | Methanothermobacter |
| OTU_146 | Archaea | Euryarchaeota | Methanobacteria | Methanobacteriales | Methanobacteriaceae | Methanobacterium |
| OTU_1482 | Archaea | Euryarchaeota | Methanomicrobia | Methanomicrobiales | Methanomicrobiales-  _incertae_sedis | Methanolinea |
| OTU_1492 | Archaea | Euryarchaeota | Methanomicrobia | Methanomicrobiales | Methanomicrobiales-  _incertae_sedis | Methanolinea |
| OTU_1504 | Archaea | Euryarchaeota | Methanomicrobia | Methanomicrobiales | Methanomicrobiales-  _incertae_sedis | Methanoregula |
| OTU_154 | Archaea | Euryarchaeota | Methanobacteria | Methanobacteriales | Methanobacteriaceae | Methanobacterium |
| OTU_1559 | Archaea | Euryarchaeota | Methanomicrobia | Methanomicrobiales | Methanospirillaceae | Methanospirillum |
| OTU_1566 | Archaea | Euryarchaeota | Methanomicrobia | Methanosarcinales | Methanosarcinaceae | Methanomethylovorans |
| OTU_1568 | Archaea | Euryarchaeota | Methanobacteria | Methanobacteriales | Methanobacteriaceae | Methanosphaera |
| OTU_1576 | Archaea | Euryarchaeota | Methanobacteria | Methanobacteriales | Methanobacteriaceae | Methanobacterium |
| OTU_1599 | Archaea | Euryarchaeota | Methanomicrobia | Methanomicrobiales | Methanomicrobiales-  _incertae_sedis | Methanoregula |
| OTU_1614 | Archaea | Euryarchaeota | Methanomicrobia | Methanomicrobiales | Methanomicrobiaceae | Methanosphaerula |
| OTU_1618 | Archaea | Euryarchaeota | Methanomicrobia | Methanomicrobiales | Methanomicrobiales-  _incertae_sedis | Methanolinea |
| OTU_1686 | Archaea | Euryarchaeota | Methanomicrobia | Methanomicrobiales | Methanomicrobiales-  _incertae_sedis | Methanolinea |
| OTU_1699 | Archaea | Euryarchaeota | Methanobacteria | Methanobacteriales | Methanobacteriaceae | Methanobacterium |
| OTU_1700 | Archaea | Euryarchaeota | Methanobacteria | Methanobacteriales | Methanobacteriaceae | Methanobrevibacter |
| OTU_1702 | Archaea | Euryarchaeota | Methanomicrobia | Methanosarcinales | Methanosaetaceae | Methanosaeta |
| OTU_1717 | Archaea | Euryarchaeota | Methanobacteria | Methanobacteriales | Methanobacteriaceae | Methanobacterium |
| OTU_1726 | Archaea | Euryarchaeota | Methanomicrobia | Methanosarcinales | Methanosaetaceae | Methanosaeta |
| OTU_1773 | Archaea | Euryarchaeota | Methanomicrobia | Methanomicrobiales | Methanomicrobiales-  _incertae_sedis | Methanolinea |
| OTU_1788 | Archaea | Euryarchaeota | Methanomicrobia | Methanomicrobiales | Methanomicrobiaceae | Methanosphaerula |
| OTU_1802 | Archaea | Euryarchaeota | Methanomicrobia | Methanomicrobiales | Methanospirillaceae | Methanospirillum |
| OTU_1812 | Archaea | Euryarchaeota | Methanomicrobia | Methanomicrobiales | Methanomicrobiales-  _incertae_sedis | Methanolinea |
| OTU_1828 | Archaea | Euryarchaeota | Methanomicrobia | Methanomicrobiales | Methanomicrobiales-  _incertae_sedis | Methanolinea |
| OTU_1855 | Archaea | Euryarchaeota | Methanomicrobia | Methanomicrobiales | Methanomicrobiales-  _incertae_sedis | Methanolinea |
| OTU_1896 | Archaea | Euryarchaeota | Methanomicrobia | Methanomicrobiales | Methanomicrobiales-  _incertae_sedis | Methanolinea |
| OTU_1898 | Archaea | Euryarchaeota | Methanomicrobia | Methanomicrobiales | Methanospirillaceae | Methanospirillum |
| OTU_1930 | Archaea | Euryarchaeota | Methanobacteria | Methanobacteriales | Methanobacteriaceae | Methanobacterium |
| OTU_195 | Archaea | Euryarchaeota | Methanobacteria | Methanobacteriales | Methanobacteriaceae | Methanobacterium |
| OTU_1965 | Archaea | Euryarchaeota | Methanomicrobia | Methanosarcinales | Methanosaetaceae | Methanosaeta |
| OTU_1979 | Archaea | Euryarchaeota | Methanomicrobia | Methanomicrobiales | Methanomicrobiales-  _incertae_sedis | Methanolinea |
| OTU_2007 | Archaea | Euryarchaeota | Methanobacteria | Methanobacteriales | Methanobacteriaceae | Methanobacterium |
| OTU_2036 | Archaea | Euryarchaeota | Methanobacteria | Methanobacteriales | Methanobacteriaceae | Methanobrevibacter |
| OTU_2046 | Archaea | Euryarchaeota | Methanobacteria | Methanobacteriales | Methanobacteriaceae | Methanobacterium |
| OTU_2122 | Archaea | Euryarchaeota | Methanomicrobia | Methanomicrobiales | Methanomicrobiales-  _incertae_sedis | Methanolinea |
| OTU_2132 | Archaea | Euryarchaeota | Methanomicrobia | Methanosarcinales | Methanosarcinaceae | Methanomethylovorans |
| OTU_2133 | Archaea | Euryarchaeota | Methanobacteria | Methanobacteriales | Methanobacteriaceae | Methanobacterium |
| OTU_2148 | Archaea | Euryarchaeota | Methanomicrobia | Methanomicrobiales | Methanospirillaceae | Methanospirillum |
| OTU_2191 | Archaea | Euryarchaeota | Methanomicrobia | Methanomicrobiales | Methanomicrobiales-  _incertae_sedis | Methanolinea |
| OTU_2218 | Archaea | Euryarchaeota | Methanomicrobia | Methanomicrobiales | Methanomicrobiaceae | Methanofollis |
| OTU_2248 | Archaea | Euryarchaeota | Methanomicrobia | Methanomicrobiales | Methanomicrobiales-  _incertae_sedis | Methanolinea |
| OTU_2278 | Archaea | Euryarchaeota | Methanomicrobia | Methanomicrobiales | Methanomicrobiales-  _incertae_sedis | Methanoregula |
| OTU_2303 | Archaea | Euryarchaeota | Methanomicrobia | Methanomicrobiales | Methanospirillaceae | Methanospirillum |
| OTU_233 | Archaea | Euryarchaeota | Methanobacteria | Methanobacteriales | Methanobacteriaceae | Methanobacterium |
| OTU_2368 | Archaea | Euryarchaeota | Methanobacteria | Methanobacteriales | Methanobacteriaceae | Methanobacterium |
| OTU_238 | Archaea | Euryarchaeota | Methanobacteria | Methanobacteriales | Methanobacteriaceae | Methanobacterium |
| OTU_2387 | Archaea | Euryarchaeota | Methanobacteria | Methanobacteriales | Methanobacteriaceae | Methanobrevibacter |
| OTU_2398 | Archaea | Euryarchaeota | Methanobacteria | Methanobacteriales | Methanobacteriaceae | Methanobacterium |
| OTU_2415 | Archaea | Euryarchaeota | Methanomicrobia | Methanomicrobiales | Methanomicrobiaceae | Methanosphaerula |
| OTU_2424 | Archaea | Euryarchaeota | Methanomicrobia | Methanomicrobiales | Methanomicrobiales-  _incertae_sedis | Methanolinea |
| OTU_2425 | Archaea | Euryarchaeota | Methanomicrobia | Methanomicrobiales | Methanomicrobiaceae | Methanosphaerula |
| OTU_2434 | Archaea | Euryarchaeota | Methanobacteria | Methanobacteriales | Methanobacteriaceae | Methanobacterium |
| OTU_2470 | Archaea | Euryarchaeota | Methanomicrobia | Methanomicrobiales | Methanomicrobiaceae | Methanosphaerula |
| OTU_2474 | Archaea | Euryarchaeota | Methanomicrobia | Methanosarcinales | Methanosaetaceae | Methanosaeta |
| OTU_2525 | Archaea | Euryarchaeota | Methanomicrobia | Methanosarcinales | Methanosarcinaceae | Methanosarcina |
| OTU_2528 | Archaea | Euryarchaeota | Methanomicrobia | Methanomicrobiales | Methanomicrobiales-  _incertae_sedis | Methanolinea |
| OTU_2562 | Archaea | Euryarchaeota | Methanomicrobia | Methanomicrobiales | Methanospirillaceae | Methanospirillum |
| OTU_2584 | Archaea | Euryarchaeota | Methanobacteria | Methanobacteriales | Methanobacteriaceae | Methanobacterium |
| OTU_2589 | Archaea | Euryarchaeota | Methanomicrobia | Methanosarcinales | Methanosaetaceae | Methanosaeta |
| OTU_2604 | Archaea | Euryarchaeota | Methanomicrobia | Methanomicrobiales | Methanospirillaceae | Methanospirillum |
| OTU_2667 | Archaea | Euryarchaeota | Methanomicrobia | Methanomicrobiales | Methanomicrobiaceae | Methanosphaerula |
| OTU_2679 | Archaea | Euryarchaeota | Methanomicrobia | Methanomicrobiales | Methanomicrobiales-  _incertae_sedis | Methanolinea |
| OTU_2683 | Archaea | Euryarchaeota | Methanomicrobia | Methanomicrobiales | Methanomicrobiales-  _incertae_sedis | Methanolinea |
| OTU_2695 | Archaea | Euryarchaeota | Methanomicrobia | Methanosarcinales | Methanosaetaceae | Methanosaeta |
| OTU_2704 | Archaea | Euryarchaeota | Methanomicrobia | Methanomicrobiales | Methanospirillaceae | Methanospirillum |
| OTU_2743 | Archaea | Euryarchaeota | Methanomicrobia | Methanomicrobiales | Methanomicrobiaceae | Methanosphaerula |
| OTU_2747 | Archaea | Euryarchaeota | Methanomicrobia | Methanomicrobiales | Methanomicrobiaceae | Methanosphaerula |
| OTU_279 | Archaea | Euryarchaeota | Methanobacteria | Methanobacteriales | Methanobacteriaceae | Methanobacterium |
| OTU_2814 | Archaea | Euryarchaeota | Methanomicrobia | Methanomicrobiales | Methanomicrobiales-  _incertae_sedis | Methanolinea |
| OTU_2846 | Archaea | Euryarchaeota | Methanomicrobia | Methanomicrobiales | Methanospirillaceae | Methanospirillum |
| OTU_2868 | Archaea | Euryarchaeota | Methanomicrobia | Methanomicrobiales | Methanospirillaceae | Methanospirillum |
| OTU_2872 | Archaea | Euryarchaeota | Methanomicrobia | Methanomicrobiales | Methanomicrobiales-  _incertae_sedis | Methanoregula |
| OTU_2878 | Archaea | Euryarchaeota | Methanomicrobia | Methanosarcinales | Methanosaetaceae | Methanosaeta |
| OTU_2901 | Archaea | Euryarchaeota | Methanomicrobia | Methanomicrobiales | Methanomicrobiales-  _incertae_sedis | Methanolinea |
| OTU_2906 | Archaea | Euryarchaeota | Methanomicrobia | Methanomicrobiales | Methanomicrobiales-  _incertae_sedis | Methanolinea |
| OTU_2937 | Archaea | Euryarchaeota | Methanobacteria | Methanobacteriales | Methanobacteriaceae | Methanobacterium |
| OTU_2944 | Archaea | Euryarchaeota | Methanomicrobia | Methanomicrobiales | Methanomicrobiaceae | Methanoculleus |
| OTU_2957 | Archaea | Euryarchaeota | Methanomicrobia | Methanomicrobiales | Methanomicrobiales-  _incertae_sedis | Methanolinea |
| OTU_302 | Archaea | Euryarchaeota | Methanomicrobia | Methanosarcinales | Methanosarcinaceae | Methanosarcina |
| OTU_3025 | Archaea | Euryarchaeota | Methanomicrobia | Methanomicrobiales | Methanospirillaceae | Methanospirillum |
| OTU_3057 | Archaea | Euryarchaeota | Methanobacteria | Methanobacteriales | Methanobacteriaceae | Methanobrevibacter |
| OTU_3066 | Archaea | Euryarchaeota | Methanomicrobia | Methanomicrobiales | Methanomicrobiales-  _incertae_sedis | Methanolinea |
| OTU_3088 | Archaea | Euryarchaeota | Methanobacteria | Methanobacteriales | Methanobacteriaceae | Methanobacterium |
| OTU_3094 | Archaea | Euryarchaeota | Methanobacteria | Methanobacteriales | Methanobacteriaceae | Methanobacterium |
| OTU_3120 | Archaea | Euryarchaeota | Methanomicrobia | Methanomicrobiales | Methanomicrobiales-  _incertae_sedis | Methanoregula |
| OTU_3123 | Archaea | Euryarchaeota | Methanomicrobia | Methanomicrobiales | Methanospirillaceae | Methanospirillum |
| OTU_3127 | Archaea | Euryarchaeota | Methanomicrobia | Methanomicrobiales | Methanocorpusculaceae | Methanocorpusculum |
| OTU_3130 | Archaea | Euryarchaeota | Methanobacteria | Methanobacteriales | Methanobacteriaceae | Methanobacterium |
| OTU_3131 | Archaea | Euryarchaeota | Methanobacteria | Methanobacteriales | Methanobacteriaceae | Methanobacterium |
| OTU_3160 | Archaea | Euryarchaeota | Methanomicrobia | Methanomicrobiales | Methanomicrobiales-  _incertae_sedis | Methanolinea |
| OTU_3208 | Archaea | Euryarchaeota | Methanomicrobia | Methanosarcinales | Methanosarcinaceae | Methanomethylovorans |
| OTU_3213 | Archaea | Euryarchaeota | Methanomicrobia | Methanomicrobiales | Methanospirillaceae | Methanospirillum |
| OTU_325 | Archaea | Euryarchaeota | Methanomicrobia | Methanomicrobiales | Methanospirillaceae | Methanospirillum |
| OTU_3289 | Archaea | Euryarchaeota | Methanomicrobia | Methanomicrobiales | Methanomicrobiales-  _incertae_sedis | Methanolinea |
| OTU_3304 | Archaea | Euryarchaeota | Methanomicrobia | Methanomicrobiales | Methanomicrobiaceae | Methanoculleus |
| OTU_337 | Archaea | Euryarchaeota | Methanomicrobia | Methanomicrobiales | Methanomicrobiales-  _incertae_sedis | Methanoregula |
| OTU_3375 | Archaea | Euryarchaeota | Methanomicrobia | Methanomicrobiales | Methanospirillaceae | Methanospirillum |
| OTU_343 | Archaea | Euryarchaeota | Methanobacteria | Methanobacteriales | Methanobacteriaceae | Methanobacterium |
| OTU_3434 | Archaea | Euryarchaeota | Methanomicrobia | Methanomicrobiales | Methanomicrobiales-  _incertae_sedis | Methanolinea |
| OTU_3464 | Archaea | Euryarchaeota | Methanomicrobia | Methanomicrobiales | Methanomicrobiaceae | Methanosphaerula |
| OTU_3470 | Archaea | Euryarchaeota | Methanomicrobia | Methanosarcinales | Methanosaetaceae | Methanosaeta |
| OTU_3500 | Archaea | Euryarchaeota | Methanomicrobia | Methanomicrobiales | Methanomicrobiales-  _incertae_sedis | Methanolinea |
| OTU_353 | Archaea | Euryarchaeota | Methanomicrobia | Methanomicrobiales | Methanomicrobiales-  _incertae_sedis | Methanolinea |
| OTU_3536 | Archaea | Euryarchaeota | Methanomicrobia | Methanomicrobiales | Methanospirillaceae | Methanospirillum |
| OTU_3580 | Archaea | Euryarchaeota | Methanomicrobia | Methanomicrobiales | Methanomicrobiales-  _incertae_sedis | Methanolinea |
| OTU_3650 | Archaea | Euryarchaeota | Methanomicrobia | Methanomicrobiales | Methanospirillaceae | Methanospirillum |
| OTU_3656 | Archaea | Euryarchaeota | Methanomicrobia | Methanomicrobiales | Methanomicrobiales-  _incertae_sedis | Methanolinea |
| OTU_367 | Archaea | Euryarchaeota | Methanomicrobia | Methanomicrobiales | Methanospirillaceae | Methanospirillum |
| OTU_3723 | Archaea | Euryarchaeota | Methanomicrobia | Methanomicrobiales | Methanomicrobiales-  _incertae_sedis | Methanolinea |
| OTU_3735 | Archaea | Euryarchaeota | Methanomicrobia | Methanosarcinales | Methanosaetaceae | Methanosaeta |
| OTU_3786 | Archaea | Euryarchaeota | Methanomicrobia | Methanomicrobiales | Methanomicrobiales-  _incertae_sedis | Methanolinea |
| OTU_3807 | Archaea | Euryarchaeota | Methanomicrobia | Methanosarcinales | Methanosaetaceae | Methanosaeta |
| OTU_3870 | Archaea | Euryarchaeota | Methanomicrobia | Methanomicrobiales | Methanospirillaceae | Methanospirillum |
| OTU_395 | Archaea | Euryarchaeota | Methanomicrobia | Methanomicrobiales | Methanomicrobiales-  _incertae_sedis | Methanolinea |
| OTU_396 | Archaea | Euryarchaeota | Methanobacteria | Methanobacteriales | Methanobacteriaceae | Methanobacterium |
| OTU_398 | Archaea | Euryarchaeota | Methanomicrobia | Methanomicrobiales | Methanospirillaceae | Methanospirillum |
| OTU_3985 | Archaea | Euryarchaeota | Methanomicrobia | Methanomicrobiales | Methanomicrobiaceae | Methanosphaerula |
| OTU_4126 | Archaea | Euryarchaeota | Methanomicrobia | Methanomicrobiales | Methanomicrobiales-  _incertae_sedis | Methanolinea |
| OTU_413 | Archaea | Euryarchaeota | Methanomicrobia | Methanomicrobiales | Methanomicrobiaceae | Methanoculleus |
| OTU_4130 | Archaea | Euryarchaeota | Methanomicrobia | Methanomicrobiales | Methanospirillaceae | Methanospirillum |
| OTU_41715 | Archaea | Euryarchaeota | Methanomicrobia | Methanomicrobiales | Methanomicrobiales-  _incertae_sedis | Methanolinea |
| OTU_418 | Archaea | Euryarchaeota | Methanomicrobia | Methanomicrobiales | Methanomicrobiales-  _incertae_sedis | Methanolinea |
| OTU_420 | Archaea | Euryarchaeota | Methanobacteria | Methanobacteriales | Methanobacteriaceae | Methanobacterium |
| OTU_422 | Archaea | Euryarchaeota | Methanomicrobia | Methanomicrobiales | Methanomicrobiaceae | Methanosphaerula |
| OTU_4221 | Archaea | Euryarchaeota | Methanobacteria | Methanobacteriales | Methanobacteriaceae | Methanobacterium |
| OTU_4316 | Archaea | Euryarchaeota | Methanomicrobia | Methanomicrobiales | Methanomicrobiales-  _incertae_sedis | Methanolinea |
| OTU_4332 | Archaea | Euryarchaeota | Methanomicrobia | Methanosarcinales | Methanosarcinaceae | Methanosarcina |
| OTU_439 | Archaea | Euryarchaeota | Methanomicrobia | Methanomicrobiales | Methanomicrobiaceae | Methanogenium |
| OTU_4402 | Archaea | Euryarchaeota | Methanomicrobia | Methanosarcinales | Methanosaetaceae | Methanosaeta |
| OTU_4422 | Archaea | Euryarchaeota | Methanomicrobia | Methanomicrobiales | Methanomicrobiaceae | Methanosphaerula |
| OTU_4439 | Archaea | Euryarchaeota | Methanobacteria | Methanobacteriales | Methanobacteriaceae | Methanobacterium |
| OTU_4533 | Archaea | Euryarchaeota | Methanobacteria | Methanobacteriales | Methanobacteriaceae | Methanobacterium |
| OTU_456 | Archaea | Euryarchaeota | Methanobacteria | Methanobacteriales | Methanobacteriaceae | Methanobrevibacter |
| OTU_4596 | Archaea | Euryarchaeota | Methanomicrobia | Methanomicrobiales | Methanomicrobiaceae | Methanosphaerula |
| OTU_4641 | Archaea | Euryarchaeota | Methanomicrobia | Methanosarcinales | Methanosaetaceae | Methanosaeta |
| OTU_4713 | Archaea | Euryarchaeota | Methanobacteria | Methanobacteriales | Methanobacteriaceae | Methanobacterium |
| OTU_4765 | Archaea | Euryarchaeota | Methanomicrobia | Methanomicrobiales | Methanomicrobiales-  _incertae_sedis | Methanolinea |
| OTU_4784 | Archaea | Euryarchaeota | Methanomicrobia | Methanosarcinales | Methanosarcinaceae | Methanosarcina |
| OTU_4789 | Archaea | Euryarchaeota | Methanomicrobia | Methanomicrobiales | Methanomicrobiales-  _incertae_sedis | Methanoregula |
| OTU_479 | Archaea | Euryarchaeota | Methanobacteria | Methanobacteriales | Methanobacteriaceae | Methanobacterium |
| OTU_4881 | Archaea | Euryarchaeota | Methanomicrobia | Methanosarcinales | Methanosarcinaceae | Methanosarcina |
| OTU_493 | Archaea | Euryarchaeota | Methanomicrobia | Methanomicrobiales | Methanospirillaceae | Methanospirillum |
| OTU_496 | Archaea | Euryarchaeota | Methanomicrobia | Methanomicrobiales | Methanospirillaceae | Methanospirillum |
| OTU_499 | Archaea | Euryarchaeota | Methanomicrobia | Methanomicrobiales | Methanomicrobiaceae | Methanosphaerula |
| OTU_508 | Archaea | Euryarchaeota | Methanomicrobia | Methanosarcinales | Methanosaetaceae | Methanosaeta |
| OTU_510 | Archaea | Euryarchaeota | Methanomicrobia | Methanomicrobiales | Methanomicrobiales-  _incertae_sedis | Methanoregula |
| OTU_515 | Archaea | Euryarchaeota | Methanomicrobia | Methanosarcinales | Methanosarcinaceae | Methanomethylovorans |
| OTU_518 | Archaea | Euryarchaeota | Methanomicrobia | Methanosarcinales | Methanosarcinaceae | Methanomethylovorans |
| OTU_520 | Archaea | Euryarchaeota | Methanomicrobia | Methanosarcinales | Methanosaetaceae | Methanosaeta |
| OTU_525 | Archaea | Euryarchaeota | Methanobacteria | Methanobacteriales | Methanobacteriaceae | Methanobacterium |
| OTU_533 | Archaea | Euryarchaeota | Methanomicrobia | Methanomicrobiales | Methanomicrobiales-  _incertae_sedis | Methanolinea |
| OTU_534 | Archaea | Euryarchaeota | Methanomicrobia | Methanomicrobiales | Methanomicrobiales-  _incertae_sedis | Methanolinea |
| OTU_549 | Archaea | Euryarchaeota | Methanobacteria | Methanobacteriales | Methanobacteriaceae | Methanobrevibacter |
| OTU_55 | Archaea | Euryarchaeota | Methanobacteria | Methanobacteriales | Methanobacteriaceae | Methanobacterium |
| OTU_562 | Archaea | Euryarchaeota | Methanomicrobia | Methanomicrobiales | Methanomicrobiales-  _incertae_sedis | Methanolinea |
| OTU_57 | Archaea | Euryarchaeota | Methanomicrobia | Methanosarcinales | Methanosaetaceae | Methanosaeta |
| OTU_590 | Archaea | Euryarchaeota | Methanobacteria | Methanobacteriales | Methanobacteriaceae | Methanobacterium |
| OTU_592 | Archaea | Euryarchaeota | Methanomicrobia | Methanomicrobiales | Methanomicrobiaceae | Methanoculleus |
| OTU_594 | Archaea | Euryarchaeota | Methanobacteria | Methanobacteriales | Methanobacteriaceae | Methanobacterium |
| OTU_612 | Archaea | Euryarchaeota | Methanomicrobia | Methanomicrobiales | Methanomicrobiales-  _incertae_sedis | Methanolinea |
| OTU_618 | Archaea | Euryarchaeota | Methanobacteria | Methanobacteriales | Methanobacteriaceae | Methanobacterium |
| OTU_638 | Archaea | Euryarchaeota | Methanomicrobia | Methanosarcinales | Methanosarcinaceae | Methanomethylovorans |
| OTU_64 | Archaea | Euryarchaeota | Methanobacteria | Methanobacteriales | Methanobacteriaceae | Methanobacterium |
| OTU_64176 | Archaea | Euryarchaeota | Methanomicrobia | Methanomicrobiales | Methanospirillaceae | Methanospirillum |
| OTU_65 | Archaea | Euryarchaeota | Methanomicrobia | Methanomicrobiales | Methanomicrobiales-  _incertae_sedis | Methanolinea |
| OTU_65920 | Archaea | Euryarchaeota | Methanobacteria | Methanobacteriales | Methanobacteriaceae | Methanosphaera |
| OTU_668 | Archaea | Euryarchaeota | Methanobacteria | Methanobacteriales | Methanobacteriaceae | Methanobacterium |
| OTU_702 | Archaea | Euryarchaeota | Methanomicrobia | Methanomicrobiales | Methanomicrobiales-  _incertae_sedis | Methanolinea |
| OTU_728 | Archaea | Euryarchaeota | Methanobacteria | Methanobacteriales | Methanobacteriaceae | Methanobacterium |
| OTU_731 | Archaea | Euryarchaeota | Methanomicrobia | Methanomicrobiales | Methanomicrobiaceae | Methanoculleus |
| OTU_745 | Archaea | Euryarchaeota | Methanomicrobia | Methanomicrobiales | Methanocorpusculaceae | Methanocorpusculum |
| OTU_746 | Archaea | Euryarchaeota | Methanomicrobia | Methanosarcinales | Methanosaetaceae | Methanosaeta |
| OTU_748 | Archaea | Euryarchaeota | Methanomicrobia | Methanosarcinales | Methanosaetaceae | Methanosaeta |
| OTU_777 | Archaea | Euryarchaeota | Methanomicrobia | Methanosarcinales | Methanosaetaceae | Methanosaeta |
| OTU_797 | Archaea | Euryarchaeota | Methanomicrobia | Methanosarcinales | Methanosarcinaceae | Methanosarcina |
| OTU_860 | Archaea | Euryarchaeota | Methanobacteria | Methanobacteriales | Methanobacteriaceae | Methanobrevibacter |
| OTU_861 | Archaea | Euryarchaeota | Methanomicrobia | Methanomicrobiales | Methanospirillaceae | Methanospirillum |
| OTU_870 | Archaea | Euryarchaeota | Methanomicrobia | Methanomicrobiales | Methanomicrobiales-  _incertae_sedis | Methanolinea |
| OTU_88 | Archaea | Euryarchaeota | Methanobacteria | Methanobacteriales | Methanobacteriaceae | Methanothermobacter |
| OTU_88466 | Archaea | Euryarchaeota | Methanomicrobia | Methanosarcinales | Methanosarcinaceae | Methanosalsum |
| OTU_90 | Archaea | Euryarchaeota | Methanobacteria | Methanobacteriales | Methanobacteriaceae | Methanobacterium |
| OTU_9042 | Archaea | Euryarchaeota | Methanobacteria | Methanobacteriales | Methanobacteriaceae | Methanosphaera |
| OTU_910 | Archaea | Euryarchaeota | Methanomicrobia | Methanomicrobiales | Methanomicrobiales-  _incertae_sedis | Methanoregula |
| OTU_915 | Archaea | Euryarchaeota | Methanomicrobia | Methanomicrobiales | Methanospirillaceae | Methanospirillum |
| OTU_916 | Archaea | Euryarchaeota | Methanomicrobia | Methanosarcinales | Methanosaetaceae | Methanosaeta |
| OTU_920 | Archaea | Euryarchaeota | Methanomicrobia | Methanomicrobiales | Methanospirillaceae | Methanospirillum |
| OTU_922 | Archaea | Euryarchaeota | Methanobacteria | Methanobacteriales | Methanobacteriaceae | Methanobacterium |
| OTU_924 | Archaea | Euryarchaeota | Methanomicrobia | Methanomicrobiales | Methanospirillaceae | Methanospirillum |
| OTU_931 | Archaea | Euryarchaeota | Methanomicrobia | Methanomicrobiales | Methanomicrobiales-  _incertae_sedis | Methanolinea |
| OTU_951 | Archaea | Euryarchaeota | Methanobacteria | Methanobacteriales | Methanobacteriaceae | Methanobrevibacter |
| OTU_96 | Archaea | Euryarchaeota | Methanobacteria | Methanobacteriales | Methanobacteriaceae | Methanobacterium |
| OTU_969 | Archaea | Euryarchaeota | Methanomicrobia | Methanomicrobiales | Methanospirillaceae | Methanospirillum |
| OTU_978 | Archaea | Euryarchaeota | Methanomicrobia | Methanomicrobiales | Methanomicrobiales-  _incertae_sedis | Methanolinea |
| OTU_986 | Archaea | Euryarchaeota | Methanobacteria | Methanobacteriales | Methanobacteriaceae | Methanobacterium |

**Table S4.** Major properties of association networks.

| Network properties | Whole | BJ | Ningbo-T | Ningbo-M | QD | Winter | Spring | Summer | Autumn |
| --- | --- | --- | --- | --- | --- | --- | --- | --- | --- |
| Threshold | 0.660 | 0.880 | 0.860 | 0.870 | 0.840 | 0.810 | 0.820 | 0.810 | 0.870 |
| Network size (n)^a^ | 104 | 338 | 320 | 308 | 425 | 120 | 124 | 150 | 165 |
| Total links | 231 | 725 | 564 | 591 | 815 | 293 | 290 | 461 | 333 |
| R^2^ of power-law^b^ | 0.753 | 0.853 | 0.932 | 0.839 | 0.897 | 0.787 | 0.832 | 0.796 | 0.852 |
| Average degree (avgK) | 4.442 | 4.290 | 3.525 | 3.838 | 3.835 | 4.883 | 4.677 | 6.147 | 4.036 |
| Average clustering coefficient (avgCC) | 0.294 | 0.224 | 0.221 | 0.267 | 0.249 | 0.410 | 0.312 | 0.371 | 0.341 |
| Average path distance (GD)^c^ | 4.888 | 5.074 | 6.912 | 6.269 | 7.055 | 4.633 | 4.303 | 4.937 | 6.132 |
| Modularity | 0.570 | 0.663 | 0.702 | 0.737 | 0.723 | 0.639 | 0.652 | 0.652 | 0.715 |
| No. of modules | 9 | 41 | 45 | 34 | 47 | 10 | 12 | 10 | 16 |
| No. of positive phage-prokaryote links | 48 | 72 | 73 | 41 | 17 | 88 | 29 | 71 | 69 |
| No. of positive prokaryote-prokaryote links | 81 | 485 | 365 | 402 | 604 | 99 | 139 | 159 | 205 |
| No. of positive phage-phage links | 42 | 5 | 50 | 19 | 44 | 37 | 43 | 111 | 13 |
| No. of negative phage-prokaryote links | 23 | 27 | 33 | 11 | 4 | 32 | 47 | 43 | 21 |
| No. of negative prokaryote-prokaryote links | 36 | 134 | 43 | 118 | 146 | 37 | 31 | 73 | 25 |
| No. of negative phage-phage links | 1 | 2 | 0 | 0 | 0 | 0 | 1 | 4 | 0 |

^a^The number of phage genes and bacterial OTUs (i.e., nodes) in the network.

^b^The square of correlation coefficient (R^2^) of the linear relationship log[P(k)] ~ -γlog(k), where P(k) is the number of nodes with k degrees (connectivity) and γ is a constant.

^c^GD: geodesic distance.**Table S5.** Dissimilarity of phage communities between months represented as β-diversity. Boldface values indicate dissimilarities between two consecutive months.

| Nov | **14.4%** |  |  |  |  |  |  |  |  |  |  |
| --- | --- | --- | --- | --- | --- | --- | --- | --- | --- | --- | --- |
| Dec | 18.4% | **16.1%** |  |  |  |  |  |  |  |  |  |
| Jan | 18.8% | 17.5% | **15.7%** |  |  |  |  |  |  |  |  |
| Feb | 21.4% | 20.8% | 19.2% | **16.6%** |  |  |  |  |  |  |  |
| Mar | 19.9% | 18.9% | 17.0% | 13.8% | **18.8%** |  |  |  |  |  |  |
| Apr | 21.1% | 21.5% | 20.2% | 19.5% | 19.1% | **18.8%** |  |  |  |  |  |
| May | 17.7% | 17.5% | 20.3% | 16.3% | 20.4% | 16.1% | **15.9%** |  |  |  |  |
| Jun | 18.6% | 18.0% | 20.7% | 18.8% | 24.7% | 16.2% | 18.0% | **13.7%** |  |  |  |
| Jul | 17.7% | 18.5% | 20.4% | 19.1% | 22.9% | 19.4% | 17.5% | 15.1% | **16.0%** |  |  |
| Aug | 18.9% | 19.4% | 19.4% | 18.8% | 20.1% | 19.0% | 18.0% | 17.1% | 18.3% | **14.2%** |  |
| Sep | 16.6% | 18.4% | 18.4% | 19.1% | 21.5% | 19.2% | 20.3% | 17.8% | 18.4% | 17.5% | **16.2%** |
|  | Oct | Nov | Dec | Jan | Feb | Mar | Apr | May | Jun | Jul | Aug |
